# A sustainability-oriented approach for performance assessment of existing buildings and a case study

**DOI:** 10.1016/j.heliyon.2024.e32151

**Published:** 2024-06-01

**Authors:** Xiaoying Wen, Dongye Zhao, Zhaoting Lv, Kainan Zhang, Yu Zhang

**Affiliations:** a Department of Architecture, Taiyuan University of Technology, Taiyuan, 030024, Shanxi, China; b Tianjin University, Tianjin, 300392, China; c Department of Civil, Construction and Environmental Engineering, San Diego State University, San Diego, CA, 92182-1324, USA; d Qilu University of Technology, Jinan, 250353, China

**Keywords:** Assessment indicator, Existing building, Fuzzy matrix assessment, Performance assessment, Sustainability, Sustainable city

## Abstract

The practice of sustainable buildings has become a general trend in urban development. Yet, with the rapid development of urban renewal, the performance of existing buildings often fails to meet the actual needs of sustainable urban development. To improve the performance of existing buildings, it is urgently needed to transform the existing structures and upgrade their functions and facilities. However, the pertinent guidelines have often been unclear for assessing the performance of existing structures, and a sounder and quantitative sustainable performance assessment approach has been lacking to guide decisions on the restoration, protection, or demolition of existing buildings. Based on a multi-expert consultation methodology, we proposed a revised flexible comprehensive assessment system and a comprehensive sustainability assessment approach. The system includes 4 categories, 7 quantitative and 47 qualitative indicators. Specifically, the assessment system involves a series of steps: 1) building a hierarchical model, 2) defining assessment indicators/items and constructing a judgment matrix, 3) determining the weight coefficients of the indicators, 4) collecting/calculating quantitative and qualitative data, 5) analyzing the data and results, and 6) drawing the assessment conclusions. A case study was presented to demonstrate the application of the approach. Compared to current practices, this comprehensive assessment system may serve as a scientifically sounder and quantitative approach to guide sustainable development of existing urban buildings.

## Highlights

• A new approach was proposed and demonstrated for assessment of existing buildings.
• Seven key sustainability indicators were identified and respective weights determined.
• Results indicate the sustainable building aspects are of utmost importance for existing buildings.
• Durability and usage performance indicators are more of the highest importance.
• The assessment system may guide sustainable utilization of existing buildings.

## 1 Introduction

The concept of sustainable city comes from the theory of sustainable development, which refers to a city that can meet sustainable needs for its residents under certain socio-economic conditions and without compromising the urban ecosystem services [1]. Accordingly, urban development and renovation are considered not only one of the root causes of non-sustainability problems, but also the opportunities of achieving improved sustainability [2]. However, the population growth and the pressure spawned by increasing demands for urban development often challenge the sustainability concept [3]. Moreover, the sustainability of urban infrastructure can be impacted by unexpected pandemics such as the COVID-19 or other disasters in terms of their economic, population, and social aspects [4]. Consequently, it is critical to 1) achieve balanced and coordinated development of urban economy [5], society, and the environment, 2) mitigate the conflicts between continuous population growth and increasing shortage of resources [6], 3) improve the quality of urban architecture, to promote urban green transformation [7], and 4) activate urban vitality [8]. Eventually, people can live in a healthier habitat accompanied with improved social, economic [9], and environmental conditions. In particular, during the global urbanization process, there are various socio-economic issues and lots of challenges due to severe resource shortage and environmental depletion in cities of various scales [[10], [11], [12]]. As cities continue to grow, many of the consolidated urban areas become targets of continuous transformations for sustainability [13,14]. The transformations also respond to the need for offering housing due to rapid growth in the urban population.

Continuous improvement of existing buildings is a key aspect of sustainability, and is regarded as one of the most effective strategies [3]. To this end, it is often inevitable to use more renewable and sustainable resources by means of retrofit or renovations. Retrofitting existing buildings offers significant opportunities for reducing global energy consumption and carbon emission [15]. This is being considered as one of the main approaches to achieve sustainability in the built environment at relatively low cost [16,17].

To promote environmental sustainability, guidelines have been published by many governments and international organizations, with recommendations on putting significant efforts towards retrofitting and reutilization of existing buildings across the world [11,18]. For instance, the International Existing Building Code has been serving as a major guideline for various organizations and members of the general public [19]. The US Federal Government provided significant financial assistance to support existing building retrofit and reuse [20,21]. In addition, the United States Code (U.S.C) and Energy Policy Act. (E.P. Act) were amended to accelerate the retrofit of the existing buildings. Likewise, the UK government published a series of codes to support existing building retrofits, such as Existing Building Energy Efficiency Standards, Green Energy and Environmental Design Pioneer Award, and Department of Energy Guidelines [22], in order to reduce the country's emissions by at least 68 % by 2030. The German Sustainable Building Council formulated the German Sustainable Building Standards [23] and a sustainable building assessment system [24], both of which have been used to guide the green construction and assessment certification of existing buildings [25]. The Japanese Government proposed the concept of green comprehensive retrofit to: 1) suppress carbon emissions, 2) facilitate performance and the comfort of the living environment, 3) promote energy conservation and emission reduction. The Singapore government provided guidelines for developers and owners to choose green building materials for construction and renovation projects, thereby driving the development of the upstream and downstream industrial chains [26]. In short, many industrialized countries have implemented various energy-efficiency measures in existing buildings with the assistance of financial aid, technical support, and policy guidance [27].

However, most of these guidelines have focused on emission reduction or energy saving. As such, a large amount of research has been carried out to develop and investigate greenhouse gas emissions or developing different standards of energy efficiency in existing buildings that will be retrofitted or refurbished [8,13,[28], [29], [30], [31]]. Yet, few studies aimed at the sustainability performance assessment and utilization of existing buildings [32,33]. To some extent, there is a natural harmony between the existing buildings and the environment, and it is linked with the climate, architecture, and people [34,35]. However, there are many uncertainties, such as how the correlation between culture, region and natural environmental factors impacts the performance assessment during the renovation process of existing buildings [36], and how to improve the sustainable development of existing buildings. Managing these uncertainties and system interactions is a substantial technical challenge for sustainable building retrofits.

From a practical aspect, the current standards and guiding principles lack a sound systematic assessment approach to guide practical application [37], and the current practice has often been relaying on personal experience and development willingness to achieve retrofit and reuse of the existing buildings [38]. On the other hand, as far as the estate enterprises are concerned, there is a lack of a scientific assessment system for the goals and technical measures [39]. Moreover, there is a lack of protection, recognition, and assessment measures for the existing building environment (such as surrounding cultural landscapes, historical customs, cultural relics, and residential environment). Therefore, it is necessary to explore scientifically sounder qualitative and quantitative indicators to guide the evaluation of existing buildings.

In China, the urban renewal construction has entered into a stage of accelerated development, and many of them are still targets of continuous transformations [40,41]. Along with the renewal and transformation of old cities, existing buildings and urban structures are experiencing physical aging and social decaying [29,42]. To a certain extent, these historical structures often fail to meet the rapidly growing material and spiritual needs of urban development and residents [43]. Due to prior local economic conditions, technical limitations, lack of necessary maintenance, and the low and obsolete retrofit standards, these existing buildings often suffer from various performance deficiencies, such as the obsolete design style, limited flexibility, and aged structure [13]. On the other hand, the structures of these buildings are generally in good conditions. Appropriate renovations can extend their economic lifespan, reduce the scale of new construction, utilize existing building resources, and minimize the associated environmental impacts [[44], [45], [46]]. As such, there is an urgent need to improve the functions and upgrade the facilities of the existing buildings [47], and it is necessary to develop a set of building sustainability criteria and rank their relative importance in order to identify the most important factors to guide the renewal of the existing buildings [48,49].

To promote industrial modernization and sustainability of urban development, the Chinese government has established the "Technical Standards for Performance Assessment of Residential Buildings" (TSPARB) [50]. The standards divide the performance into five aspects: applicability, environmental impacts, economic, safety and endurance. Yet, the standards were mainly aimed to existing residential buildings. For the performance assessment of other types of existing buildings, e.g., hospital, school, and office buildings, the relevant qualitative and quantitative indicators need to be further researched and tested. In particular, the protection, identification and assessment measures are lacking in the social and environmental aspects (such as surrounding cultural landscapes, historical customs and customs, cultural relics, and regional areas).

To ensure a comprehensive and science-based assessment of the performance of existing buildings, we need to address the following critical issues, 1) develop a set of qualitative and quantitative assessment indicators, 2) improve the environmental quality of existing buildings in accord with the concept of "sustainable cities", and 3) improve and make best of the performance conditions of existing buildings. These outstanding problems can be solved through comprehensive renovation, development, and utilization. To this end, scientific and effective assessment methods and assessment criteria must be formulated, which can provide scientifically sounder assessment of existing buildings and guidance for urban decision-makers [51].

This study aimed to develop a systematic sustainability-oriented performance assessment approach for existing buildings, which suits the local and regional context of China, as well as to establish a way to assess the relative importance of the key sustainability criteria through a weighted categorization approach. Specifically, we aimed to develop scientifically sounder approaches to 1) categorize the performance data of existing urban buildings based on the fuzzy matrix theory, 2) integrate qualitative and quantitative factors and establish key assessment criteria based on experts’ consultation, 3) analyze and rank the relative importance of the different factors, and 4) improve the reliability, accuracy and objectivity of the assessment results using a progressive hierarchical decision-making structure model.

## 2 Literature review of existing, leading rating systems, and determination of key assessment criteria

Various index systems have been developed to evaluate the conditions of buildings worldwide [[52], [53], [54], [55]]. Table S1 in the Supplementary Materials (SM) lists some of the major rating systems that have been widely used for assessing new and/or existing buildings, including BREEAM (adopted by European countries), LEED (America, Europe, and Asia region, e.g., Korea Republic, India, China, etc.), BEAM Plus (Hong Kong) and Green Star (Australia, New Zealand, and Africa) [56]. These systems have different certification schemes and conditions.

In China, TSPARB was first published in 2005 by the Ministry of Housing and Urban Rural Development, and then revised in 2022. However, TSPARB only aimed for assessing the housing quality of domestic buildings. In 2019, Xu et al. [57] proposed a six-parameter system for the assessment of demolition of existing buildings (ADEB), i.e., service performance, economic impact, social identity, local development, building location, and building safety. Yet, the ADEB rating system was focused on the demolition of the existing buildings, but ignored some other important factors, such as performance value, conservation, and retrofit, which. Furthermore, the ADBE system did not take into account the individual characteristics of buildings and the extrinsic indicators.

In general, the current rating systems of existing buildings use 5–10 key indicators as the level 1 criteria (Table 1). In addition, key sustainability indicators are identified by certain criteria to the sustainability performance of buildings, including economic, social, environmental, and other aspects of sustainable development.

**Table 1:** Commonly used level 1 assessment criteria and distribution of the credit points for existing buildings [56,58].

	BREEAM 2015 International refurbishment (151 points)	BEAM Plus Existing Buildings v2.0 (315 points)	ASGREB 2015 China (1000 + 100 point)	TSPARB 2022 China (1000 point)
1	Management (20)	Management (44+5B)	Basic score for prerequisite items (400)	Usage (250)
2	Health and wellbeing (22)	Site aspects (49+2B)	Safety and durability (100)	Environment (250)
3	Energy (34)	Materials and Waste Aspects (53+2B)	Health and wellbeing (100)	Economy (200)
4	Transport (11)	Energy use (51+2B)	Occupant Convenience (100)	Safety (200)
5	Water (9)	Water use (41 + 14B)	Resources saving (200)	durability (100)
6	Materials (14)	Indoor environmental quality (50+2B)	Environment livability (100)	
7	Waste (13)	Innovative techniques (27B)	Promotion and innovation (100)	
8	Land use and ecology (5)			
9	Pollution (13)			
10	Innovation (10)			

It is noteworthy that all the four rating tools in Table 1 consider the environment, economy, and society as three pillars of the sustainable development. Furthermore, the sustainability assessment criteria are divided into three aspects and twelve categories. The three aspects refer to the economic, social, and environmental indicators of buildings, whereas the twelve categories include: usage, management, safety, durability, health and wellbeing, environment, indoor environmental quality, land use and ecology, occupant convenience, economy (energy saving, water efficiency, materials, waste, etc.), carbon emission, and innovation techniques.

Based on our broad review of the literature and a wide range of assessment practices and through expert consultation using the FAHP tool, we prioritized 12 assessment criteria (see below). The expert consultation was carried out through a series of carefully designed questionnaires as described in Section 3.2. Such an approach was expected to offer more accurate weighting of the assessment factors and more thorough and sounder assessment of given buildings.

## 3 Methodology and data collection

**Fig. 1:**
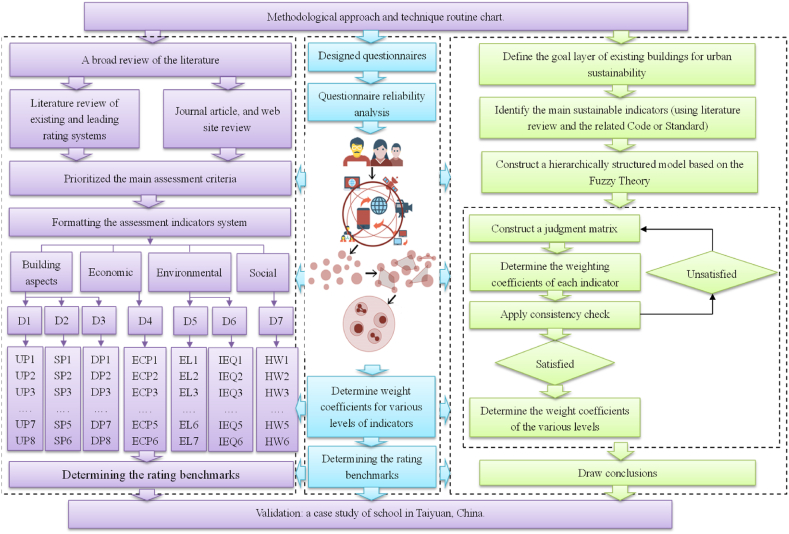
Methodological routine and process workflow for the sustainability performance assessment of existing buildings.

Fig. 1 illustrates the progressive hierarchical assessment approach to assess the performance of existing buildings towards sustainable urban development. The approach consists of the six specific steps: 1) Constructing a hierarchically structured model; 2) Establishing the key assessment criteria; 3) Formulating a judgment matrix; 4) Determining the weighting coefficients of each indicator; 5) Calculating the outcome in a hierarchical manner; and 6) Drawing the conclusions and providing performance assessment comments.

The process started with a thorough literature analysis and a desktop review of pertinent guidelines and technical notes on existing buildings. These reviews and analysis served as the foundation for identifying and establishing the key indicators. Secondly, we employed a well-designed experts’ consultation method, where a series of questionnaire were distributed to selected experts in the relevant construction building and construction fields from five major representative cities, China. And then, based on the feedback and analysis, the four-level indicators and weight coefficient of each level were determined via the FAHP method. Lastly, a sustainability assessment model of existing building was formulated. Subsequently, the application of the model was demonstrated through a case study.

### 3.1 The fuzzy analytic hierarchy process (FAHP)

The FAHP is an extension of the analytic hierarchy process (AHP) under the fuzzy environment, which is used for evaluation of multi-criteria alternatives under the fuzzy environment known as fuzzy multi-criteria decision making (FMCDM) [59]. FAHP analyzes an FMCDM structure by constructing a hierarchical model, with its factors classified according to different assessment factors and determined through the hierarchical calculation approach, which is based on the fuzzy theory and decision-makers’ opinions [52]. FAHP is also considered as the best fuzzy multicriteria decision making (FMCDM) method [56]. This method has been widely adopted for its ease of use, systematic problem structuring, and ability to calculate both criteria weights and alternative priorities. It addresses the imprecision in the AHP by replacing exact numbers with fuzzy numbers, which represent the linguistic expressions in fuzzy AHP. This approach accommodates vague judgments by assigning membership degrees to exact numbers, indicating the extent to which these numbers align with an expression [60].

This approach is one of the multi-criteria decision-making tools used in practice for solving problems involving several criteria and sub-criteria, which are acquired based on expert judgments [53,54,61]. A five-point scale (based on the Saaty fundamental scale) is used to measure the relative preferences of the decision criteria [52,54,62], and the weights are calculated for each factor based on pairwise comparisons.

Numerical variables are analyzed and judged by the semantic subdivision [63,64] and the Likert scale to determine the hierarchical level of each factor. First, we prioritized and simplified the variables to seven criteria based on survey of experts’ opinions, available codes or standards [65,66], and previous research findings on the existing buildings [48,49,56,61,[67], [68], [69], [70], [71], [72], [73], [74], [75], [76], [77], [78], [79], [80], [81], [82], [83], [84], [85], [86]], then identified the key assessment criteria, assigned the score-weights to different structures, and established a performance certification and grading system.

### 3.2 Questionnaire design and expert consultation

It is worth noting that the other well-established building rating systems, such as, BREAM, LEED, and BEAM have also adopted questionnaires and interviews to develop credit points for their rating systems [15,[87], [88], [89]]. In our work, we developed a two-part questionnaire design approach for collecting information on these indicators. The first part includes the demographic information of all respondent experts, i.e., personal details, education level, their professions, and working experience in the construction industry; the second part aims to identify the order of significance of the various performance dimensions of the existing buildings, i.e., the 12 categories given above: 1) Ranking position of the given 12 categories; 2) Determination of the significance of each sub-indicator of the assessment scheme; 3) Determine weight coefficients for various levels of indicators.

The questionnaire was used to evaluate each index on a fundamental FAHP linguistic scale of judgment, which indicates five levels of importance. The voters were invited to score each item according to the degree of importance, with 1 indicating "equal unimportant", 2 ″moderate important", 3 ″strong important", 4 ″very strong important", and 5 ″extreme important".

**Fig. 2:**
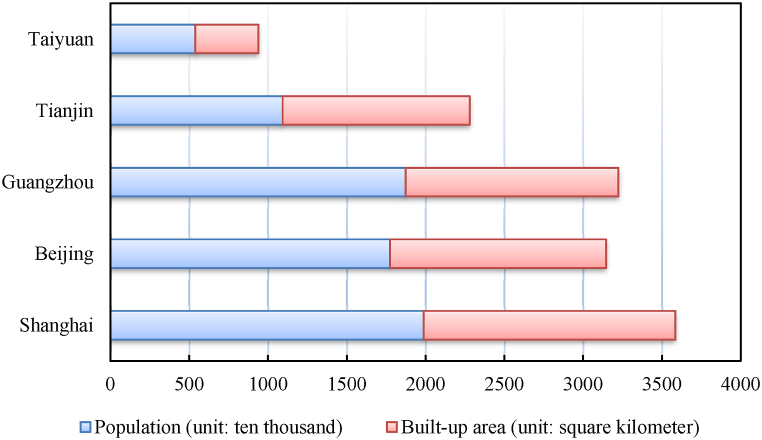
Population and built-up area of the five major cities (data from Government Website).

The expert survey was administered through on-site questionnaires, emails of invitation letters, and online interviews over a six-month period from January to June of 2023 in five major cities in China: Beijing, Taiyuan, Guangzhou, Tianjin, and Shanghai City. Beijing, Guangzhou, Tianjin, and Shanghai are the megacities of China, with a population being over 10 million and the urban built-up area having undergone tremendous growth (Fig. 2), whereas Taiyuan is a typical provincial capital city in China. The cities are located across a large area from the north to the coastal south and south east of China. Therefore, these cities may represent the well-developed major cities of China. As the urban population continues to grow, the urbanization in these cities continues to increase, so as the pressure on the shortage of resources and the environment challenges. In addition, these cities house the most talents and experts in the construction filed, which are essential for the assessment.

To be comprehensive, a total of 74 valid answers from experts in the construction industry were collected from the on-site and online interviews. The results were presented in terms of both relative (%) and absolute (n) frequencies (see Table 2). The number of answers is higher than those reported by others. For instance, Assefa et al. solicited a total of 32 experts’ opinions [90], Ali and Al Nsairat [91] reached out to a total 60 consultants, and Mahmoud obtained 20–40. In addition, we also considered diversity of the expert group to represent multiple stakeholders, educational levels, ages, experience, and cultural backgrounds.

**Table 2:** Demographic information of all respondent experts.

Description	Number of experts	Percentage (%)
**Gender**		
Male	41	55.4 %
Female	33	44.6 %
**Total**	74	100 %
**Profession or occupation**		
University Professors	12	16.2 %
Project Managers	7	9.5 %
Urban Planners	6	8.1 %
Architecture	16	21.6 %
Structural Engineers	12	16.2 %
Drainage Designers	3	4.1 %
HVAC Engineers	3	4.1 %
Electrical Engineers	3	4.1 %
Decoration Engineers	3	4.1 %
Economist	5	6.8 %
Government official	4	5.4 %
**Total**	74	100 %
**Years of working experience of experts in construction industry**		
≤5 years	4	5.4 %
6–10 years	5	6.8 %
11–15 years	15	20.3 %
16–20 years	25	33.8 %
21–25 years	14	18.9 %
26–30 years	7	9.5 %
＞30 years	4	5.4 %
**Total**	74	100 %
**Education level of experts**		
Bachelor degree	33	44.6 %
Master degree	13	17.6 %
Doctor degree	23	31.1 %
Others	5	6.8 %
**Total**	74	100.0 %
**Have you involved in the assessment decision?**		
Yes	39	52.7 %
No	35	47.3 %
**Total**	74	100.0 %

**Fig. 3:**
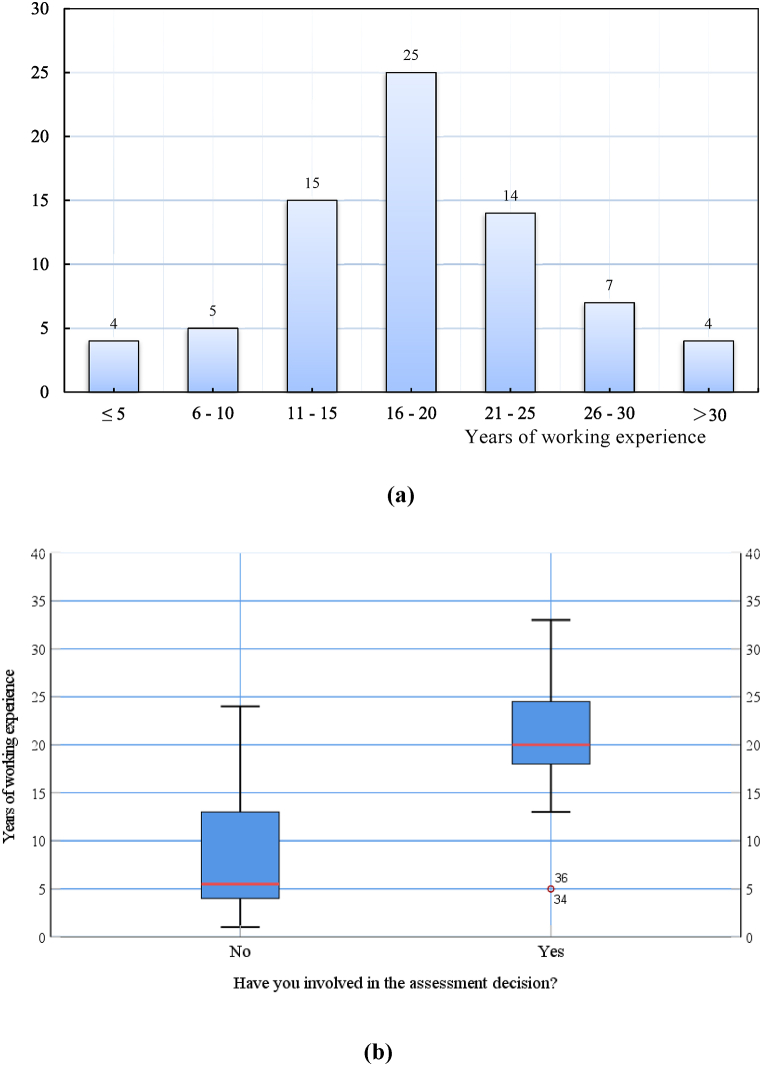
**(a)** Years of working experience of questionnaire respondents in construction industry, and **(b)** Years of working experience of questionnaire respondents as assessment decision makers.

Of the 74 experts, 55.4 % (n = 41) were males, and 44.5 % (n = 33) females. The mean age was 39.6 (SD: 1.125, range: 30–55 years old). The professions or occupation covered various fields of the construction industry and multi-stakeholders. Furthermore, the average years of working experience of participating experts were 17.32 (SD: 9.73, range: 3–45 years). Moreover, sixty-five (or ∼ 82.4 %) experts had 11–30 years of working experience (Fig. 3a), ∼48.6 % had an MS or Ph.D. degree, and ∼52.7 % had some experience of assessment decision (see Fig. 3b).

### 3.3 Questionnaire reliability analysis

The Cronbach's alpha (α) has been commonly employed for assessing response reliability, with values spanning from 0 to 1. A higher Cronbach's α than 0.8 signifies enhanced internal consistency and data reliability, while an α value of >0.6 is considered acceptable [64,90]. In this study, the Cronbach's α value of the total samples was 0.958, which was calculated using the software SPSS, indicating that the results of the questionnaire survey were robust and reliable.

## 4 Results and discussion

### 4.1 Formatting the assessment indicators

The average overall score of the sorting indicators was calculated based on the ranking of all respondents' opinions via Equation (1):

(1) Average overall score =(Σ frequency × weight) / number of the respondent experts

When optimizing the main indicators, the weighting factors were determined based on the ranking position of a given assessment parameter. For example, if there are three options involved, the weight for the first place is 3, and that for the second place is 2, and so on.

**Fig. 4:**
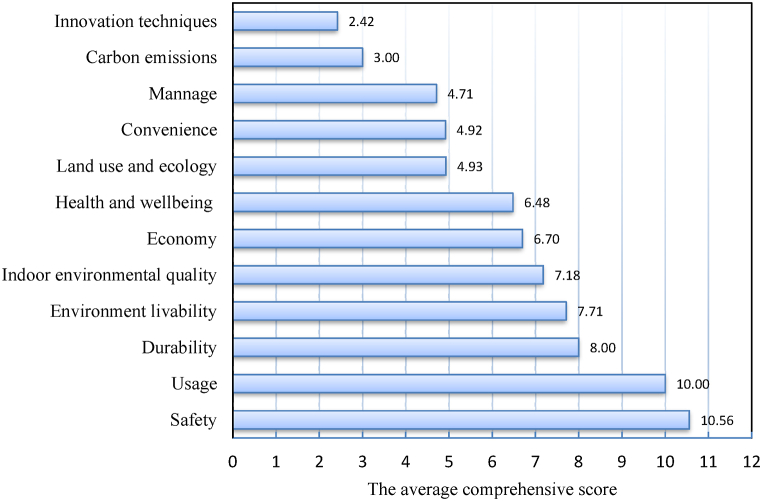
The average scores of the respondent experts with respect to 12 assessment indicators.

As shown in Fig. 4, safety was the most important indicator for the existing buildings, the average score was 10.56, followed by usage (10.00) and durability (8.00). The average value for all the 12 parameters was 6.38 (A) (SD: 0.727, range: 2.42–10.56). These results indicate the significance levels of each indicator for the target buildings. Given that the values of “land use and ecology”, “convenience”, “manage”, “carbon emissions”, and “innovation techniques” were <5.0, these five indicators were treated as sub-indicators, whereas the other 7 as the main key indicators, which are designated as “usage performance (UP)”, “safety performance (SP)”, “durability performance (DP)”, “economy (saving water, saving land, saving material and energy use) (ECP)”, “environmental livability (EL)”, “indoor environmental quality (IEQ)”, and “health and wellbeing (HW)”.

Furthermore, the participating experts were solicited to select sub-indicators from a range of items found in various codes or standards [19,50,87,88,92] and through an extensive literature review [15,24,26,27,[32], [33], [34], [35], [36],^41^,^43^,^58^,^89^,[93], [94], [95], [96], [97]].

The experts were also asked to rate the level of significance of the sub-indicators using a five-point scale (“negligible”, “optional”, “common”, “required”, and “mandatory”). The classification criteria were: 1) if ≥ 50 % of the experts rate a third-level indicator as “mandatory”, then it is classified as a “mandatory” item; 2) if ≥ 50 % of the experts rate an indicator as “common” or “required”, it is then classified as “required”; and 3) if ＜30 % of the experts rate an indicator as “optional” or “negligible”, the indicator is “negligible”.

Based on these classification rules and upon consultation with the 74 experts (from the built environment across five major Chinese cities: Beijing, Taiyuan, Guangzhou, Tianjin, and Shanghai City), forty-seven third-level indicators were classified as “required”, and thirteen (13) indicators as “mandatory”. No indicator was classified as “negligible” (see Table S2 in the SM).

Then, credit points (CPs) were established for each layer of indicators through collaboration with these 74 invited experts, who were entrusted to assign credit point scores to the sub-indicators, and then the indicators were weighted average. Pertinent information elucidating the importance of allocating credit points to each indicator was shared with the invited experts, supplemented by a guidance derived from the preceding four-stage review process.

Upon further data analysis and consultation with the experts, some of the categories were merged, added, or removed. Consequently, the final categories turned out to be 4 first-level sustainable categories (building aspects [90], economic, environmental, and social) and 7 s-level indicators (see Table S3 in the SM). The 7 s-level indicators are usage performance (UP), safety performance (SP), durability performance (DP), economy (ECP), environmental livability (EL), indoor environmental quality (IEQ), and health and wellbeing (HW), which affect the sustainability pillars either directly or indirectly.

**Fig. 5:**
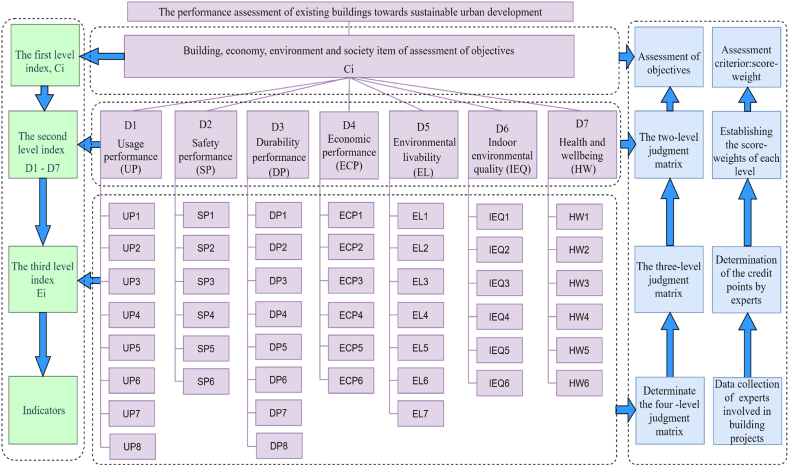
The hierarchical structure of performance assessment of existing buildings.

Based on these main level indicators and sub-indicators, a hierarchal structure essential for assessment was developed (Fig. 5), which sets the target goal (i.e., sustainability performance assessment of existing buildings) at the top and defines the four levels of indicators. Such a flexible comprehensive assessment scheme is expected to be especially useful in China and likely other developing countries.

### 4.2 Determine weight coefficients for various levels of indicators

**Fig. 6:**
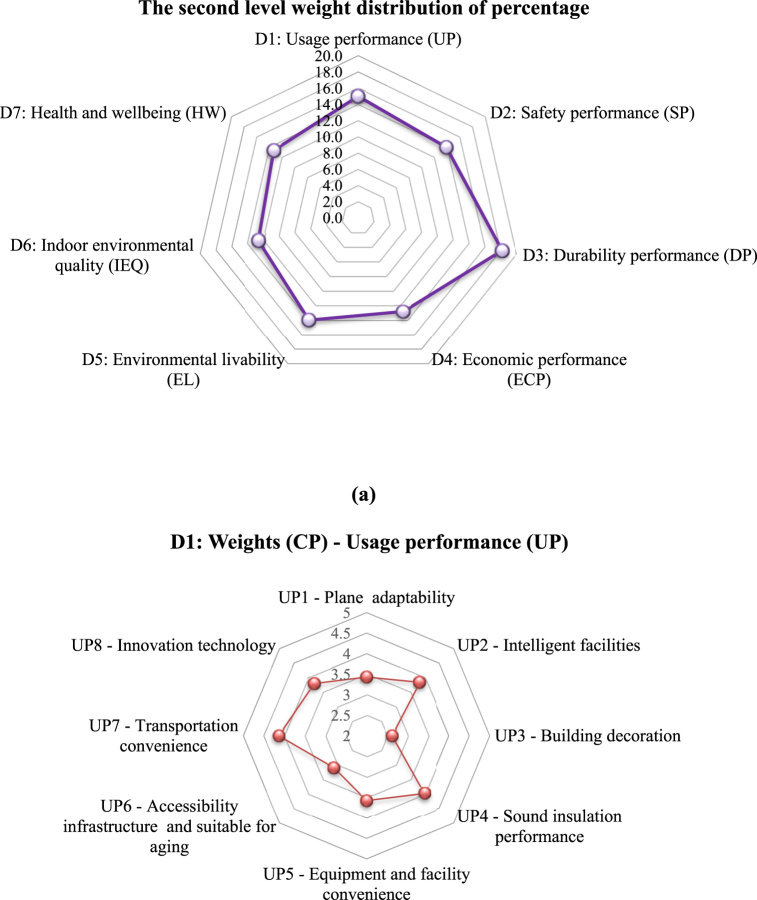

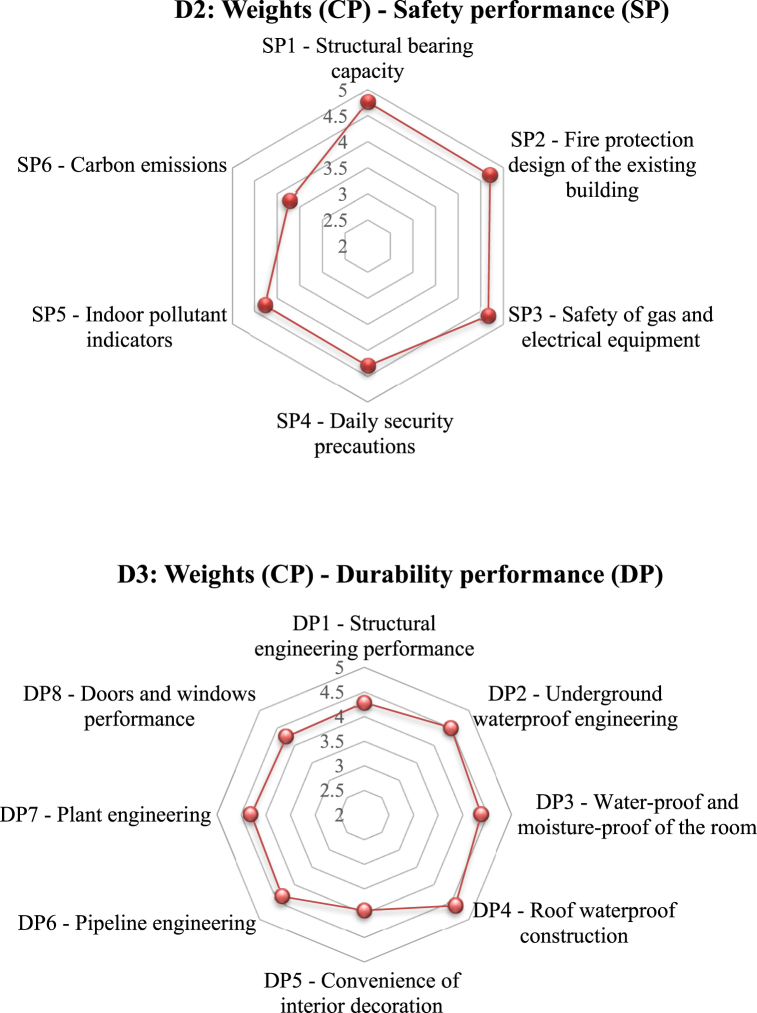

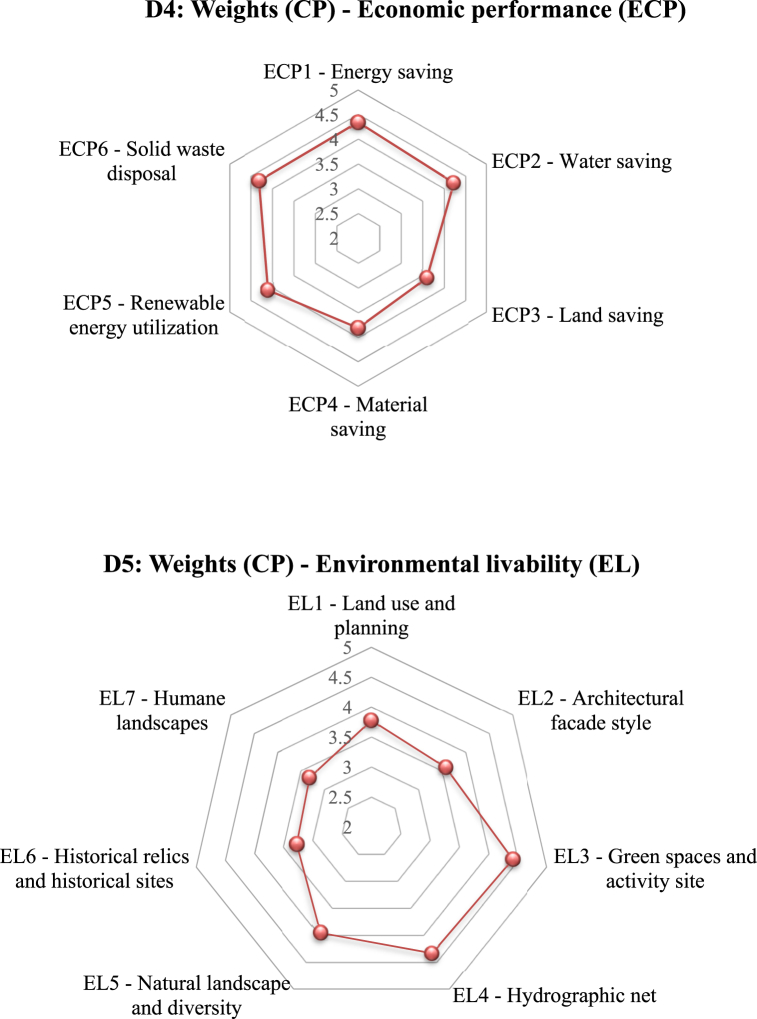

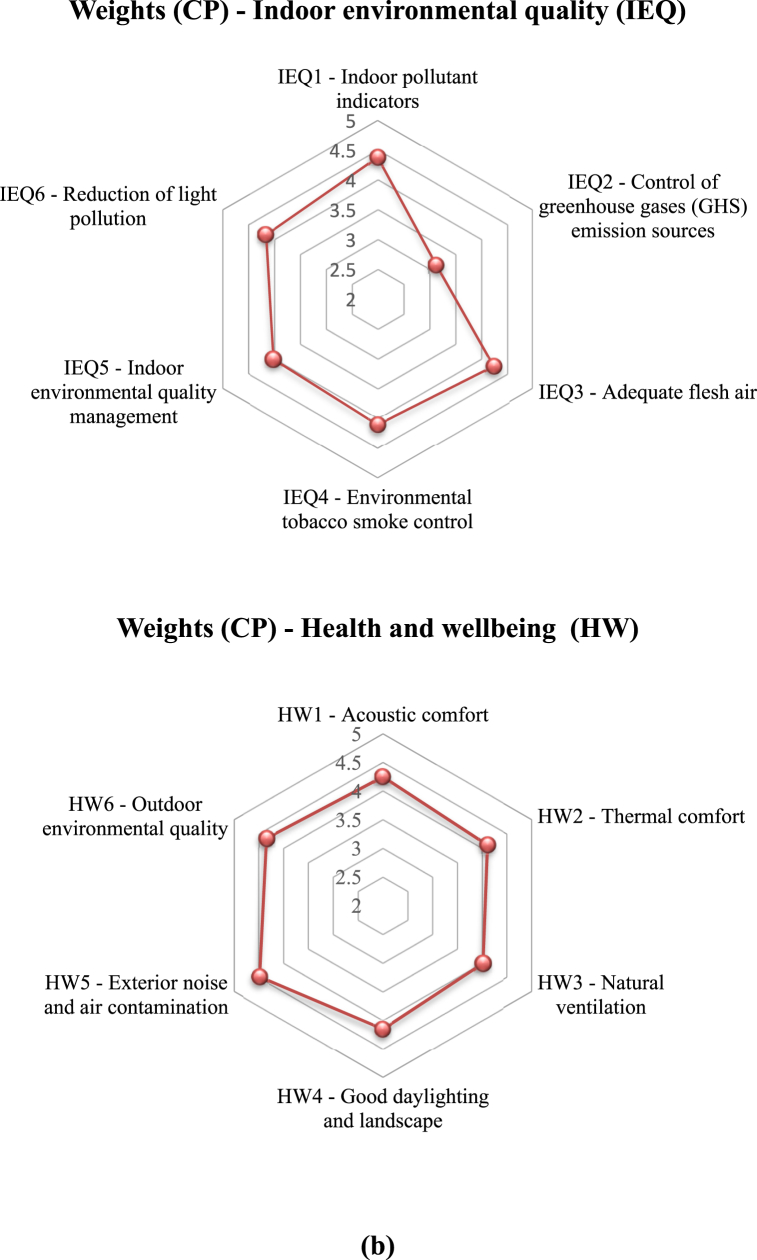

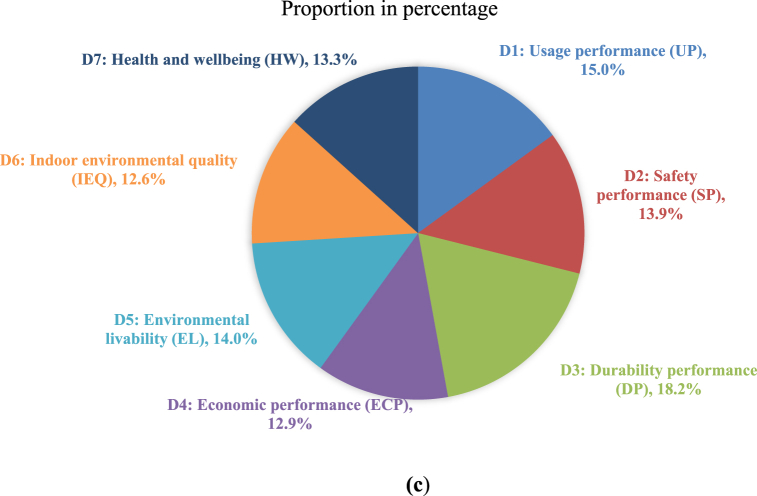
**(a)** Radar chart of credit points (CP) distribution for each second level indicator; **(b)** Radar chart of CP distribution for each third level indicator, and **(c)** Percentage of each key indicator of D1 to D7.

Table S4 in the SM lists the mean credit points assigned based on the five-point scale for the third-level indicators. Furthermore, Fig. 6a gives the distribution of weighting scores for each second-level indicator (D1 through D7). The total credit points (CPs) for each index were determined by summing up the CPs of the respective third-level indicators (Equations (2), (3))).

(2) $$W_{\text{CP}}=\frac{\sum VS}{n}$$

(3) $$W_{Z}\left(D1\right)=\text{Weight}\;\left(\text{CP}\right)=\sum W_{CP}\left(D1,D2,D3,\ldots \text{Dn}\right)$$

where ∑*VS* *=* sum of the credit points (W_CP_) of each of the third-level indicators given by the invited experts, n = the total number of the invited experts, W_CP_ = CPs of the third-level indicators, and W_Z_ = weight score of the indicators.

For example, the Wz (D1) for the second-level index “usage performance” is 28.51 (see Table S4), which is the summation of the values of its seven indicators (UP1 = 3.43, UP2 = 3.84, UP3 = 2.62 UP4 = 3.99, UP5 = 3.58, UP6 = 3.11, and UP7 = 4.14). Table S4 also reveals that “durability performance (DP),” “usage performance (UP)”, “environmental livability (EL)”, and “safety performance (SP)” (W_DP_ = 34.63 CP; W_UP_ = 28.51 CP; W_EL_ = 26.69 CP; and W_SP_ = 26.5 CP) are important indexes for the performance assessment of retrofit and utilization of existing buildings.

The weights of distribution analyzed from the expert consultation varied for the second-level indicators (Fig. 6a). The key indicators such as “durability performance” (18.2 score) and “usage performance” (15.0 score) received higher weightages from the experts. The results also indicate that the building aspects (47.1) were considered more essential and important than other three main pillars (environmental, social, and economic) [52,83]. Moreover, these results also show that the sustainable building aspects are of utmost importance for the overall performance assessment of the existing buildings and for aiding in improving reuse and enhancing overall health and quality of life. The results agree with the findings by Mahmoud et al. [98]. In addition, the indicators like “indoor environment quality” (12.6) and “economy” (12.9) received a lower weightage. Although these weighting values vary from one country to another, it is deemed that the relative weighting ranking in the different regions tends to be the same [33,42,83,84,96]. In general, the final weights of the main indicators follow the sequence of: durability > usage > environmental livability > safety > health and being > indoor environmental quality > economic performance.

It is crucial to determine the ranking of each assessment indicator according to the significance of the scores for the third level indicators. Fig. 6b shows the radar charts of the credit point distribution for each third-level indicators (D1 – D7).

Under the usage performance (D1, UP) indicator, the transportation convenience (UP7) and sound insulation performance (UP4) received a weighting value of 4.14 and 3.99, respectively. According to the expert survey, the higher weight values of these two sub-indicators were associated with the importance of the location for both transportation and the usage of the existing buildings.

For the safety performance (D2, SP) indicator, the highest weights were assigned to the structural bearing capacity and fire protection design, with values of 4.77 and 4.72, respectively. These sub-indicators are considered most essential in the performance assessment of the existing buildings because of the critical roles of structural stability and fire protection in the usability of existing buildings [99]. In contrast, the other sub-indicator “carbon emissions” received a lower weightage with a value of 3.74.

Under the durability performance (D3, DP) indicator, the visual roof waterproof construction (DP4) and water-proof and moisture-proof (DP3) were rated of the highest interest, with values of 4.62 and 4.38, respectively, whereas the convenience of interior decoration (DP5) received a lower weight of 3.95. Hence, the waterproof conditions are considered essential.

On the economy aspect (D4, ECP), the energy saving factor had the highest weight of 4.35, whereas the solid waste disposal, water saving, and renewable energy utilization also received high weights (4.32, 4.24, and 4.11, respectively), while material saving and land saving were considered of lower importance (3.82 and 3.61).

With respect to the environmental livability indicator (D5, EL), green spaces and activity site and hydrographic net got the highest weights of 4.42 and 4.34, respectively; in contrast, the sub-indicators of the historical relics and historical sites and humane landscape received lower values of 3.28 and 3.32, respectively.

As for the IEQ indicator (D6), the highest weights were given to the indoor pollutants and adequate fresh air factor, with the weighting values of 4.38 and 4.25, respectively; whereas the values of reduction of light pollution and environmental tobacco smoke control were 4.16 and 4.11, respectively, and the control of greenhouse gases (GHS) emission sources received the lowest weighting (3.14).

For the health and wellbeing indicator (D7), the exterior noise and air contamination and outdoor environmental quality factors acquired the highest weights 4.49 and 4.35, respectively; and the acoustic comfort and good daylighting and landscape sub-indicators obtained comparable weight values (4.25 and 4.16, respectively). According to the expert survey, the weighting sequence resulted from heavier considerations of the health impacts of the indoor air qualities and exterior and interior noise pollution. While the differential weighting may vary by locations, it agrees with the findings of Mahmouds’ [98].

The overall assessment performance of existing buildings (P_1, …7_) was then calculated via

(4) $$P_{1,\ldots 7}=\frac{W_{CP}\left(D\right)}{\sum_{1}^{7}W_{CP\;}\left(D\right)}X100\%$$

where $\sum_{1}^{7}W_{CP\;}\left(D\right)$ *refers to* summation of the credit points of each index of the indicators (W_CP_), and $W_{CP}\left(D\right)$ is the summation of the credit points of each index of the sub-indicator (W_CP_).

Fig. 6c gives the percentage of each key indicator (D1 - D7), which also reflects a balance among the indicators. Indicators D3 (18.2 %), D1 (15.0 %) and D5 (14.0 %) were regarded by the experts as the main criteria that should be given the highest priority in the assessment of an existing building performance. The assessment criteria may differ for the different rating systems established in different countries, such as LEED, BREEAM, BEAM, Green Mark, GREB Chinese, TSPARB Chinese, etc.

### **4.3** Scoring third-level items and determining the weighting factors

The third-level weight index system involved a voting process by the 74 experts on the importance of each assessment index. The voters were invited to score each item according to the degree of importance, with 1 indicating "very unimportant", 2 ″not important", 3 ″normal", 4 ″important", 5 ″very important".

Taking together the questionnaires gives the weighting factors of E_i_. The importance degree coefficients (a_i_) of factor E_i_ were calculated via

(5) $$a_{i}=\sum_{j=1}^{n}a_{ij}/\sum_{j=1}^{n}\left(\sum_{i=1}^{m}a_{ij}\right),\left(i=1,2,L,m\right)$$

Likewise, the first-level weights for the 7-item indicator set,

(6) $$C_{1}=\{D_{1},D_{2},D_{3}\},C_{2}=\{D_{4}\}{,C}_{3}=\{D_{5},D_{6}\},C_{4}=\{D_{7}\}$$

were obtained according to the percentage and by normalizing the first-level index system:

$$A_{2j}=\left\{0.15,0.139,0.182,0.12.9,0.14,0.12.6,0.133\right\}$$

$$A_{1j}=\{0.471,0.129,0.266,0.133\;\}$$

### 4.4 Determining the rating benchmarks

**Fig. 7:**
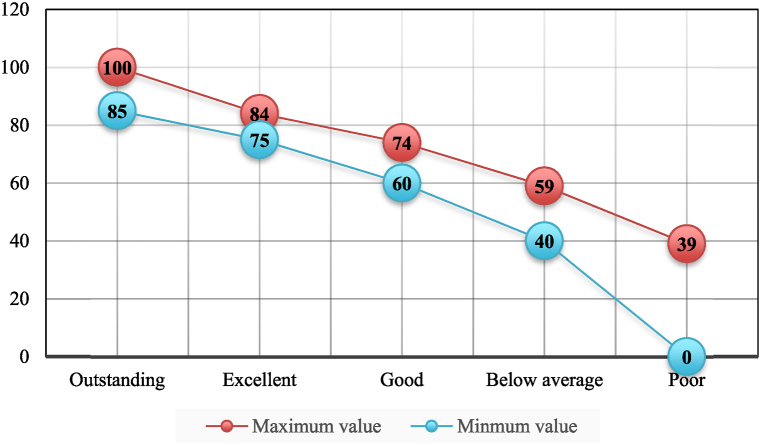
The maximum and minimum score values for the individual performance levels in accord with the five-grade rating system.

As mentioned above, several internationally popular green building evaluation systems have adopted the multiple rating level benchmarks, such as BREEAM (6 levels), LEED (4 level), BEAM Plus (4 level), BSAM (6 levels), EEWH (3 levels), and ESGB (3 levels) (see Table 3). We adopted a similar approach to obtain the numerical scores for each rating level from the experts, i.e., the maximum and minimum values for each level of assessment of the objectives, which must be in the range of 0 and 100. Then, we determined the five performance levels (outstanding, excellent, good, below average, and poor). Fig. 7 provides the score values for the individual performance levels.

**Table 3:** Rating system benchmarks adopted from the popular green building evaluation systems.

Rating system worldwide	Rating level benchmarks	Score thresholds (%)
**LEED**	**4**	
	Platinum	≥80
	Gold	60–79
	Silver	50–59
	Certified	40–49
**BREEAM**	**6**	
	Outstanding	≥85
	Excellent	70–84
	Very good	55–69
	Good	45–54
	Pass	30–44
	Unclassified	＜30
**BEAM Plus**	**5**	
	Excellent	70 %
	Very good	60 %
	Good	50 %
	Satisfactory	40 %
	Unsatisfactory	＜40 %
**BASM**	**6**	
	Outstanding	≥82
	Excellent	73–81
	Very good	63–72
	Good	55–62
	Acceptable	40–54
	Unclassified	＜39
**ESGB (2019 version)**	**3**	
	Three-star	≥85
	Two-star	70–84
	One-star	60–69
	Unclassified	＜59
**TSPARB (2023 version)**	**3**	
	3A	≥85
	2A	75–84
	1A	60–74
	Unclassified	＜59

## 5 Constructing the judgment matrix

As aforementioned, the judgment matrix was divided into a four-level structure (Fig. 5). The first level index (C_i_) consists of building item (C1), economy (C2), environment (C3), and society (C4), where C1 corresponds to usage, safety, and durability, C2 to energy, water, land, material, and waste, C3 to environmental livability and indoor environmental quality, and C4 to health and wellbeing. The second-level index (D_i_) includes 7 items as listed in Section 4.1. The third level (Ei) include 47 items as defined in Table S3 in the SM. The target layer is B (defined in the performance assessment of existing buildings towards sustainable urban development).

### **5.1** The third-level judgment matrix

The third-level judgment matrix was determined based on scoring the various indicators of the existing buildings by the 74 experts, who evaluated each of the third-level indicators as V1, V2, V3, V4, and V5 to form a numeric vector corresponding to each judgment. If there are n_ij_ households where the experts judged u_i_ as level V_j_ by n experts, then the n_ij_ experts form a hierarchical assessment vector fuzzy set R_i_ for all levels of the index assessment.

(5) $$R_{i}=\left(\frac{n_{i1}}{n},\frac{n_{i2}}{\begin{array}{c} n \end{array}},\ldots ,\frac{n_{ip}}{n}\right)=\left(V_{i1},V_{i2}\ldots ，V_{ip}\right)\;\left(p=1,2,3,4,5\right)$$

Then the single-factor assessment matrix of the three-level comprehensive assessment is:

(6) $$E_{i}=A_{\text{ij}}*R_{i}=A_{3j}*\left[\begin{array}{cccc} V_{11} & V_{12} & \ldots & V_{1p}\\ V_{21} & V_{22} & \ldots & V_{2p}\\ \ldots & \ldots & \ldots & \ldots \\ V_{i1} & V_{i2} & \ldots & V_{ip} \end{array}\right]\;\left(i=1,2,\ldots ,n,p=1,2,3,4,5\right)$$

### **5.2** The second-level judgment matrix

The corresponding relationship between the second-level D_ij_ and the third-level E_i_ data sets is:

D1={E1,E2,E3,E4,E5,E6,E7,E8}

D2={E9,E10,E11,E12,E13,E14}

D3={E15,E16,E17,E18,E19,E20,E21,E22}

D4={E23,E24,E25,E26,E27,E28}

D5={E29,E30,E31,E32,E33,E34,E35}

D6={E36,E37,E38,E39,E40,E41}

D7={E42,E43,E44,E45,E46,E47}

Based on the relationship, a third-level comprehensive assessment is carried out on D_ij_, and the single-factor assessment standard set is:

(7) $$\text{Di}=\text{Aij}*\text{Ei}=A2j*\left(E_{i1},E_{i2},\ldots ,E_{\text{iP}}\right)\;\left(I=1,2,\ldots ,n,p=1,2,3,4,5\right)$$

### **5.3** The first-level judgment matrix

The relationship between the first-level C_ij_ and the second-level Di data set is:

C1={D1,D2,D3}

C2={D4}

C3={D5,D6}

C4={D7}

According to the relationship, a first-level comprehensive assessment is conducted on C_ij_, and the single-factor assessment standard set is:

(8) Ci=Aij*Di=A1j*(Di1,Di2,…,Dip)(i=1,2,…,4,p=1,2,3,4,5)

(9) Ci=Aij*Di=A1j*,(i=1,2,…,4,p=1,2,3,4,5)

### **5.4** Assessment target of objectives

The corresponding relationship between the first-level C_ij_ and the goal-level B_i_ data set is:

(10) $$B=\left\{C_{1},C_{2,}\;C_{3},C_{4}\right\}$$

The sub-vector of the target top-level can then be obtained via:

(11) S=A*Ci=[B1B2B3B4B5]

where B is the final membership vector, and A is the weight of each index.

Then, a comprehensive assessment was performed via:

(12) S=B*J

where J is the corresponding rating benchmark vector (85, 75, 60, 40, 0) of the assessment set, i.e., outstanding, excellent, good, below average, and poor. The specific score was obtained through matrix multiplication to determine the overall performance of a given existing building.

According to the weights and assessment values of indicators at all levels, the performance assessment results of existing buildings were obtained. The higher the score, the better the performance of the existing building, and the higher the potential value of renovation and development. The following assessment case criteria were followed.

Case 1 The corresponding score is between 85 and 100. The building performance is excellent; it carries extremely high social, historical, and cultural value and is strictly protected.

Case 2 The score is between 75 and 84. The building performance is good; it has certain social, historical, and cultural value, and belongs to the category of buildings that should be protected.

Case 3 The score is between 60 and 74. The building performance is moderate; it has a certain use value, and it is recommended to renovate and reuse.

Case 4 The score between 40 and 59. The building performance is poor; it has little or no social, historical, and cultural value and use value, and should be demolished.

Case 5 The score is less than 40. The building performance is very poor; it has no value and should be demolished.

## 6 Formulation of assessment model and pre-assessment: a case study

According to our preliminary on-site investigation of selected middle school campuses in Taiyuan City, Shanxi Province, Taiyuan Foreign Language Middle School was selected as a model assessment site in this study. The goal was to perform an in-depth investigation of the environment inside and outside the campus and a comprehensive assessment of the existing building performance of the teaching buildings.

**Fig. 8:**
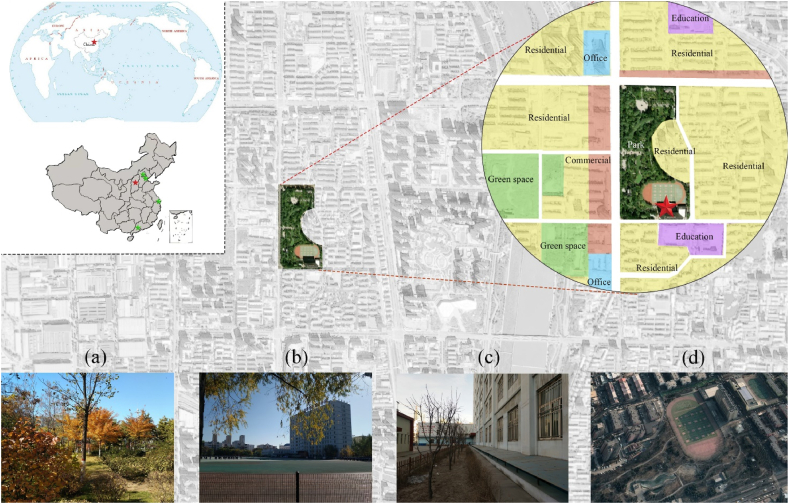
Location of the case of school, Taiyuan, China. **(a)** The green environment, **(b)** The main teaching building and the sport field, **(c)** The façade of the teaching building, and **(d)** The campus map (from Google map).

The school is located at the intersection of Yongle South Street and Qianfeng North Road, Wanbailin District, Taiyuan. It is adjacent to a major public park (Yifen Park), forming a garden-like integrated landscape (Fig. 8a–c), covering an area of 29 acres. The campus includes a 12-storey main teaching building (9228.5 m^2^, built in 2009) (Fig. 8c–d), the Shaw Complex Building (7400 m^2^, built in 2000), and a standard 400-m plastic sport field on the north side of the main teaching building.

According to the preliminarily established assessment index system, a quantitative analysis was carried out with respect to the four indicators, i.e., the building layer, the economy layer, the environmental layer, and the society layer. In addition, building upon the in-depth investigation of the site and the assessment of the quantitative indicators of the collation level of the objective data of the internal and external environments, the assessment of the qualitative indicators was conducted through the campus questionnaires of teachers and students during the break between classes.

**Fig. 9:**
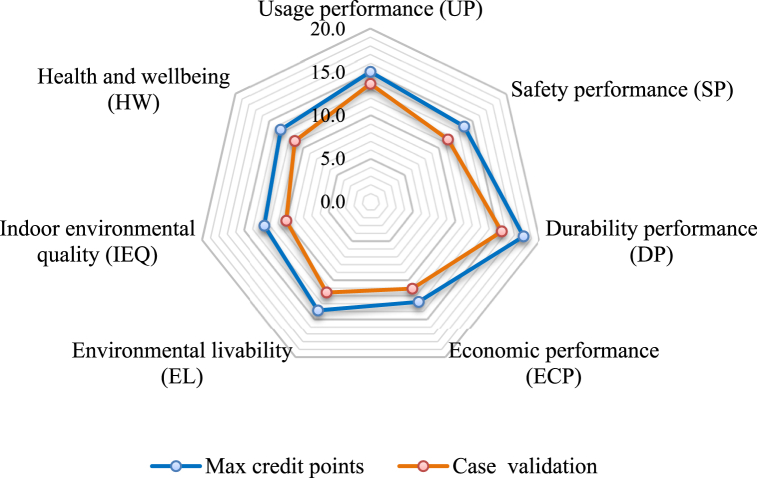
Radar chart of credit points (CP) distribution for the sustainability performance assessment of an existing building in Taiyuan, China.

According to the indicator weights and scores of 157 users (teachers and students conducted vector scoring and voting on each indicator of Table S3, a mean value was then obtained (Table S5). Fig. 9 illustrates the credit point distribution of the existing building case and max credit points. In this indicator system, the weight scores follow the order of: DP (15.6) > UP (13.6) > EL (11.7) > SP (11.5) > HW and ECP (11.2) > IEQ (10.0). Because of the increase in the user's demand for living comfort and the environmental conditions, the corresponding weights were larger and were close to that for safety.

The final score S was calculated to be 84.8. Therefore, the teaching building was judged to have excellent architectural performance and possess certain ecological, social, historical and cultural value, and thus should be well preserved.

## 7 Comparison with other rating systems and discussion on strengths and limitations

### 7.1 Comparative analysis with existing rating systems

**Fig. 10:**
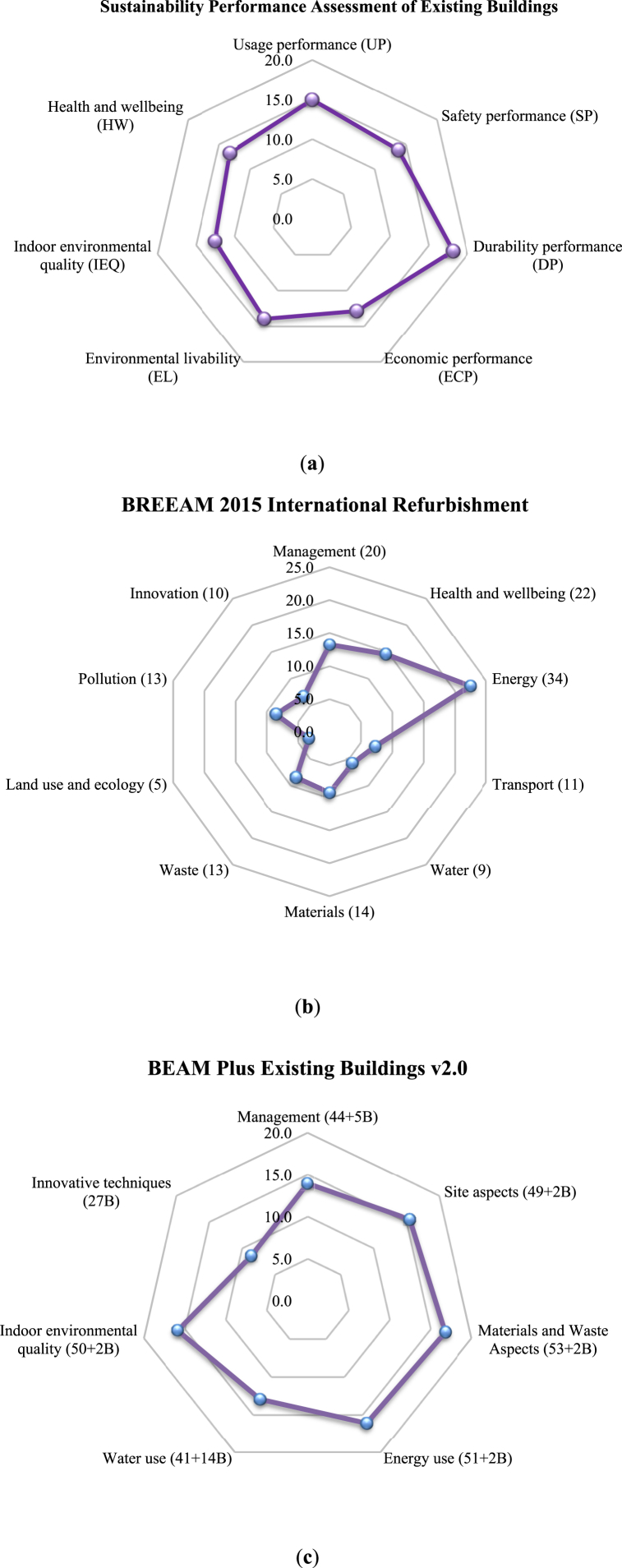

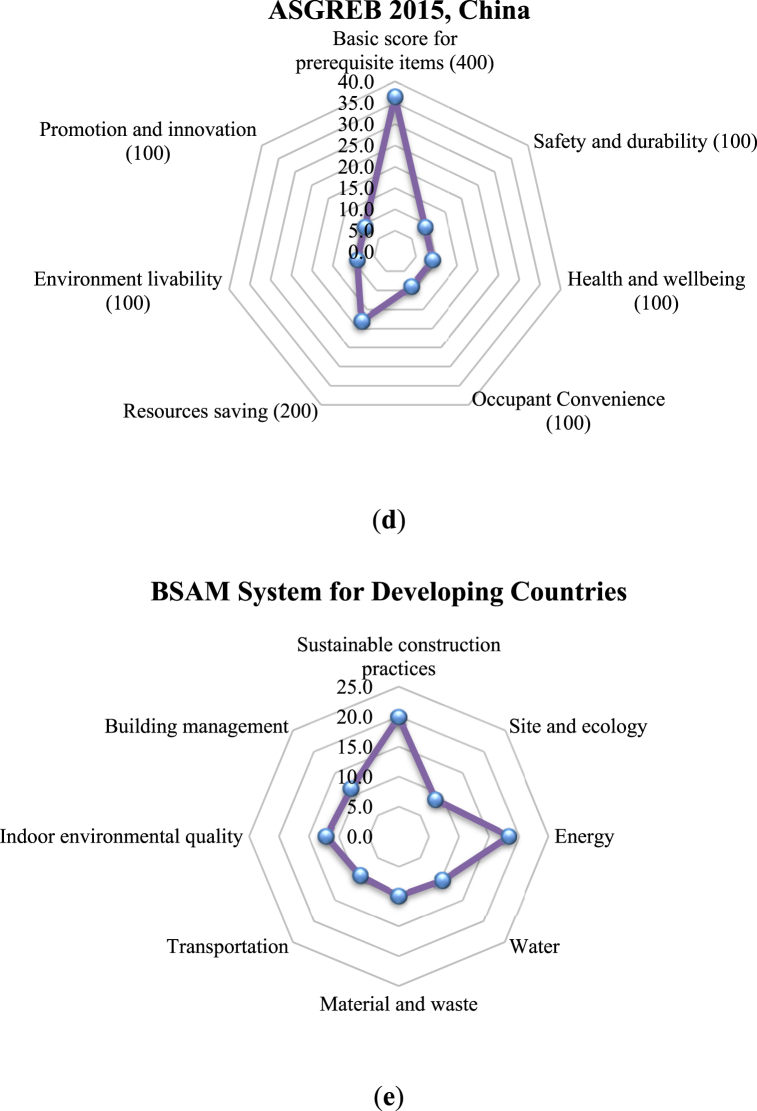
Radar charts of the credit points percentage (%) distribution of the key indicators using five different assessment systems: **(a)** Sustainability Performance Assessment of Existing Buildings; **(b)** BREEAM 2015 International Refurbishment; **(c)** BEAM Plus Existing Buildings v2.0; **(d)** ASGREB 2015, China; and **(e)** BSAM System for developing countries.

This section compares the proposed sustainability performance assessment model with four commonly used rating tools of the latest versions for assessing existing buildings. Fig. 10 presents the radar charts of the credit points distribution (%) of the key indicators (Fig. 10a − Sustainability Performance Assessment of Existing Buildings, Fig. 10b − BREEAM 2015 International Refurbishment, Fig. 10c − BEAM Plus Existing Buildings v2.0, Fig. 10d − ASGREB 2015, China, and Fig. 10e − BSAM System for developing countries).

Fig. 10 indicates significant differences in the key indicators and percentages of each indicator among the rating tools. Several sustainability indicators, such as “health and wellbeing” and “IEQ”, are used in some of the rating tools, and to some extent, there are similarities among “economy”, “energy”, “water”, and “material and waste” indicators. In addition, most of the existing rating tools include “Sites aspect (land use and ecology)”, “water”, “energy”, and “materials and waste”, which is different from the proposed sustainability performance assessment approach, wchere “water”, “energy”, and “materials and waste” are sub-indicators under “economy”. Moreover, the proposed assessment system showed a more profound role of the indicators: “durability performance (18.2 %)”, “usage performance (15.0 %)”, “safety performance (13.9 %). This again shows the new approach underscores the significance of sustainability aspects in the overall performance assessment of existing buildings, which is similar to the sustainable construction practices” criterion (19.91 %) in the BSAM system. Hence, the new tools stresses that there is an urgent need to improve reuse and sustainability of existing buildings.

### 7.2 Strengths and limitations of the sustainability performance assessment approach

The revised comprehensive approach integrates multiple-dimensions of sustainability, including safety, durability, usage, economy, environmental livability, indoor environmental quality, and health and wellbeing. The assessment system can serve as a decision-making platform for ecological restoration, protection, and/or demolition of existing urban buildings. It provides a scientifically sounder and more comprehensive approach for evaluating existing building performance with respect to the various pillar aspects of sustainability.

It is noted that the classification rules including the assessment indicators, sub-indicators, and weighting factors were built upon the situations from five major Chinese cities, which may not fully capture the complexity and nuances of sustainable urban development in other cities or regions. Moreover, while 74 well-selected experts represent a reasonable size and diverse expertise, cautions should be exercised in the selection of the experts to assure data quality and representativeness. Further validation is needed by testing the evaluation system to different types of existing buildings in different geographic locations.

Based on the FAHP, the hierarchical assessment model allows for a systematic evaluation of existing building performance, which can help identify areas for improvement and guide decision-making in urban development. Combining both qualitative and quantitative indicators allows for a more holistic evaluation of existing building performance, which can help capture the multifaceted nature of sustainability and provide a standardized assessment framework. It will contribute to the broader goal of creating more sustainable and livable urban environments. However, incorporating fuzzy sets into the AHP complicates the calculation process due to the existence of multiple fuzzy sets and the complexity of the associated operations.

The revised assessment approach was targeted on assessing sustainability performance of existing buildings, but there is also a need to evaluate the performance of new buildings for sustainable urban development. It would be interesting to modify the approach and compare the performances for new and existing buildings. This could include expanding the sample size and conducting more empirical studies.

### 7.3 Implications for sustainable urban development

To facilitate field application of the evaluation system, further validation and pilot-testing of the system is needed to gauge and improve the applicability and adaptability to broader regional conditions as well as adjust/optimize the system parameters towards diverse end users. To this end, an Excel-based framework can be developed, which accommodates the hierarchical assessment structure including Assessment Preparation, Data Collection, Scoring and Score Analysis, Indicator Weighting, Overall Rating, and Decision Making. The computer package will facilitate entering and analysis of the assessment parameters (the first-level items, key sustainability indicators, and sub-indicators) and calculation of the overall scores and weights. To maintain long-term evaluation consistency and accuracy, a stable and large pool of experts from diverse stakeholders and sectors should be established. The assessment results should be compared with other currently used tools, and results be judged by a broad pool of experts and practitioners and sustainability-related regulatory personnel. Continuous improvement of the assessment tool should be carried to keep up with the rapidly evolving urban development.

Urban existing buildings are very complex due to different ages, structure style, surroundings, and scale of building. Owing to regional and cultural differences, obtaining basic data can be difficult, and thus, it is often necessary to add more flexible evaluation index variables and levels. Moreover, there is a need to explore more reasonable and accurate weight distribution methods to improve the reliability of evaluation results. Lastly, the applicability to new construction projects needs to be explored.

## 8 Concluding remarks

Enhancing the performance of existing buildings is a pivotal element and one of the most efficient strategies for sustainable urban development. Yet, a scientifically sounder and practically more reliable approach has been sought to assess the sustainability-oriented performance of existing buildings. To address this research and practical need, this study provided an improved "elastic assessment index system" for evaluating urban existing building performance towards sustainable urban development. The major findings are summarized as follows.

1) A progressive hierarchical assessment approach was constructed to assess the existing building performance towards sustainable urban development based on a four-step process.
2) Four first-level items on the sustainable development, seven key sustainability indicators, and 47 sub-indicators of the performance assessment of existing buildings were identified and the respective weights were assigned based on the analysis of the data collected via expert’ consultations.
3) The percentage of the weighting scores for each sustainability items was calculated. The first level items including “society” (13.3 score), “environment” (26.6 score), and “economy” (12.9 score) received a lower weightage than the building aspects (47.1 %). This approach for performance assessment of existing buildings placed more emphasis on the building aspect compared to the social and economic parameters of sustainability.
4) The results also indicate that the sustainable building aspects are of utmost importance for the overall performance assessment of the existing buildings and their associated consequences and for aiding in improving reuse and enhancing overall health and quality of life, which also differs from major existing rating systems.
5) In general, the final weights of the main indicators follow the sequence of: durability > usage > environmental livability > safety > health and wellbeing > indoor environmental quality > economic performance.
6) In addition, we established the certification grading system scales: outstanding, excellent, good, below average, and poor. A case study of performance assessment was performed using a high school teaching building as a model to demonstrate the assessment approach.

This assessment system takes into account both qualitative and quantitative indicators and is based on a set of thoroughly designed and itemized questionnaire and scores from experts representing the various sectors/aspects of the construction field. Hence, it reduces the bias and generality of current assessment practices, allowing for a sounder and more comprehensive analysis of existing buildings. It may serve as a decision-making platform for the ecological restoration, protection, and/or demolition of existing urban buildings. The new approach may also guide the assessment of the value, individual building performance, environmental sustainability in the process of urban renewal and renovation.

Given the complex and dynamic nature of the assessment process as well as geographical differences, and the assessment system can be adjusted and optimized according to the specific case conditions.

## Declaration of ethics statement

Participants and the university research committee both gave their consent by ethical standards (Approval committee: The Academic Sub Committee of College of Architecture, Taiyuan University of Technology. Approval Number: 0516/2023).

## Data availability statement

No data associated with this study has been deposited into a publicly available repository. Additional data will be available upon request.

## CRediT authorship contribution statement

**Xiaoying Wen:** Writing – review & editing, Writing – original draft, Methodology, Formal analysis, Conceptualization. **Dongye Zhao:** Supervision. **Zhaoting Lv:** Validation, Investigation. **Kainan Zhang:** Investigation. **Yu Zhang:** Data curation.

## Declaration of competing interest

The authors declare that they have no known competing financial interests or personal relationships that could have appeared to influence the work reported in this paper.
