# Supplementary material for: A sustainability-oriented approach for performance assessment of existing buildings and a case study
Source: Heliyon. 2024 Jun 1;10(12):e32151. doi: 10.1016/j.heliyon.2024.e32151 (PMC11341241; doi:10.1016/j.heliyon.2024.e32151)
Supplement: Multimedia component 1 [file mmc1.docx]

**A Sustainability-Oriented Approach for** **Performance Assessment** **of Existing Buildings** **and a Case Study**

*Xiaoying Wen**^1,3^*,* *Dongye Zhao**^2,^* **,* *Zhaoting Lv^4^, Kainan Zhang^1^, Yu Zhang^1^*

*^1^Department of Architecture*

*Taiyuan* *University of Technology, Taiyuan 030024, Shanxi, China*

*Corresponding authors Email:* wenxiaoying@tyut.edu.cn

*^2^Department of Civil, Construction and Environmental Engineering*

*San Diego State University, San Diego, CA 92182-1324*

*Corresponding authors Email:* *[dzhao2@sdsu.edu](mailto:dzhao2@sdsu.edu) (D. Zhao)*

*^3^Tianjin University, Tianjin 300392, China*

*^4^* *Qilu University of Technology, Jinan 250353, China*

**Supplementary Materials**

Tables: 5

Appendixes: 3

**Table S1.** Major existing green rating systems worldwide.

| Existing rating systems | Countries or regions | Version of years | New buildings | Existing buildings |
| --- | --- | --- | --- | --- |
| BREEAM | Europe (United Kingdom, Croatia, Germany, Netherlands, Poland, Spain, Sweden etc.) | 1990  2015  2022 etc. | √ | √ |
| BEAM Plus | Hong Kong | 1996, 2016(version 2.0) | √ | √ |
| LEED | Europe (Germany, Turkey, Spain, Poland, Sweden, Italy), America (United States of America, Canada), Asia (China, Korea Republic, India etc.) | 1998  2000  2009  2013 (version 4) | √ | √ |
| CASBEE | Japan | 2001 | √ | √ |
| Green Mark  TSPARB | Singapore  China | 2005, 2011  2005, 2022 | √  √ | √ |
| ESGB | China | 2006, 2014, 2019 | √ |  |
| Green Star | Oceania (Australia, New Zealand) | 2008, 2009, 2011 | √ |  |
| DGNB | Germany | 2007, 2018 | √ |  |
| ASEB  ASGREB | United States of America  China | 2013  2015 |  | √  √ |
| BSAM | Sub-Saharan Africa | 2020 | √ | √ |

Sources: Various websites and literatures (Rosa and Haddad [52], Olawumi et al. [53] and Assefa et al. [83])

Notes: BREEAM, Building research establishment environmental assessment

BEAM Plus, Building environmental assessment method

LEED, Leadership in energy and environmental design

CASBEE, Comprehensive assessment system for built environment efﬁciency

TSPARB, Technical standard for performance assessment of residential buildings

ESGB, Evaluation standard for green building (China)

DGNB, Deutsche Gesellschaft für Nachhaltiges Bauen (German)

ASEB, Assessment standard for green building

ASGREB, Assessment standard for green retrofitting of existing building

BSAM, building sustainability assessment method

BSAS, building sustainability assessment system

**Table S2.** Determination of the significance of each third–level indicator of the assessment scheme.

| Third level indicator | Negligible  n (%) | Optional  n (%) | Common  n (%) | Required  n (%) | Mandatory required  n (%) | Inference |
| --- | --- | --- | --- | --- | --- | --- |
| **Usage performance (UP)** | | | | | | |
| UP1 | 0 | 2.7 | 28.4 | 39.2 | 29.7 | R |
| UP2 | 1.4 | 5.4 | 17.6 | 59.5 | 16.2 | R |
| UP3 | 0 | 2.7 | 31.1 | 43.2 | 22.9 | R |
| UP4 | 0 | 1.4 | 10.8 | 36.4 | 51.4 | MR |
| UP5 | 0 | 1.4 | 10.8 | 41.9 | 45. | R |
| UP6 | 0 | 2.7 | 10.8 | 47.3 | 39.2 | R |
| UP7 | 0 | 0 | 20.3 | 45.9 | 33.8 | R |
| UP8 | 5.4 | 9.5 | 47.3 | 20.3 | 17.6 | R |
| **Safety performance (SP)** | | | | | | |
| SP1 | 1.4 | 0 | 2.7 | 12.1 | 83.8 | MR |
| SP2 | 1.4 | 0 | 1.4 | 20.2 | 77.0 | MR |
| SP3 | 0 | 1.4 | 1.4 | 25.7 | 71.6 | MR |
| SP4 | 0 | 2.7 | 8.1 | 44.6 | 44.6 | R |
| SP5 | 0 | 1.4 | 10.8 | 45.9 | 41.9 | R |
| SP6 | 2.7 | 4.1 | 29.7 | 43.2 | 20.3 | R |
| **Durability performance (DP)** | | | | | | |
| DP1 | 0 | 1.4 | 13.5 | 40.5 | 44.6 | R |
| DP2 | 0 | 2.7 | 5.4 | 31.1 | 60.8 | MR |
| DP3 | 0 | 2.7 | 9.5 | 8.1 | 52.7 | MR |
| DP4 | 0 | 1.4 | 5.4 | 23.0 | 70.3 | MR |
| DP5 | 0 | 1.4 | 23.0 | 55.3 | 20.3 | R |
| DP6 | 0 | 1.4 | 12.2 | 35.1 | 51.4 | MR |
| DP7 | 0 | 4.1 | 12.2 | 33.8 | 50.0 | MR |
| DP8 | 0 | 2.7 | 8.1 | 51.4 | 37.8 | R |
| **Economic performance (ECP)** | | | | | |  |
| ECP1 | 0 | 2.7 | 9.5 | 37.8 | 50.0 | MR |
| ECP2 | 0 | 2.7 | 8.1 | 51.4 | 37.8 | R |
| ECP3 | 1.4 | 1.4 | 20.3 | 43.2 | 32.4 | R |
| ECP4 | 0.0 | 0.0 | 18.9 | 60.8 | 20.3 | R |
| ECP5 | 1.4 | 1.4 | 16.2 | 47.3 | 33.8 | R |
| ECP6 | 1.4 | 1.4 | 9.5 | 39.2 | 48.6 | R |
| **Environmental livability (EL)** | | | | | | R |
| EL1 | 2.7 | 5.4 | 24.3 | 45.9 | 21.6 | R |
| EL2 | 4.1 | 5.4 | 33.8 | 40.5 | 16.2 | R |
| EL3 | 0 | 1.4 | 6.8 | 40.5 | 51.4 | MR |
| EL4 | 0 | 0 | 16.2 | 33.8 | 58.1 | R |
| EL5 | 0 | 2.7 | 25.7 | 44.6 | 27.0 | R |
| EL6 | 10.8 | 8.1 | 40.5 | 23.0 | 27.0 | R |
| EL7 | 12.2 | 6.8 | 33.8 | 31.1 | 16.2 | O |
| **Indoor environmental quality (IEQ)** | | | | | | |
| IEQ1 | 0 | 1.4 | 12.2 | 35.1 | 51.4 | MR |
| IEQ2 | 16.2 | 16.2 | 21.6 | 29.7 | 16.2 | R |
| IEQ3 | 0 | 2.7 | 10.8 | 47.3 | 39.2 | R |
| IEQ4 | 0 | 4.1 | 14.9 | 47.3 | 33.8 | R |
| IEQ5 | 0 | 0 | 18.9 | 60.8 | 20.3 | R |
| IEQ6 | 0 | 0 | 14.9 | 45.9 | 35.1 | R |
| **Health and wellbeing (HW)** | | | | | | |
| HW1 | 1.4 | 2.7 | 6.8 | 47.3 | 41.9 | R |
| HW2 | 0 | 1.4 | 18.9 | 47.3 | 32.7 | R |
| HW3 | 0 | 1.4 | 17.6 | 51.4 | 29.7 | R |
| HW4 | 1.4 | 2.7 | 18.9 | 32.4 | 44.6 | R |
| HW5 | 1.4 | 1.4 | 9.5 | 23.0 | 64.9 | MR |
| HW6 | 1.4 | 2.7 | 9.5 | 32.4 | 54.1 | R |

Note: for column “inference” – R is required; MR is mandatory required.

**Table S3.** Key decision support index systems used to assess conditions of the performance assessment of the existing buildings.

| Item number (IN) C_i_ | The first level index (score points of percentage) | IN (score points of percentage) | The second level index  D_i_ | Indicators of the third level  E_i_ | Credit points (CP) |
| --- | --- | --- | --- | --- | --- |
| C1 | Building aspects  (47.1) | W_Z_ (D1) = 15 | Usage performance  (UP) | UP1 - Plane adaptability | 1.8 |
|  |  |  |  | UP2 - Intelligent facilities | 2.0 |
|  |  |  |  | UP3 - Building decoration | 1.4 |
|  |  |  |  | UP4 - Sound insulation performance | 2.1 |
|  |  |  |  | UP5 - Equipment and facility convenience | 1.9 |
|  |  |  |  | UP6 - Accessibility infrastructure and suitable for aging | 1.6 |
|  |  |  |  | UP7 - Transportation convenience | 2.2 |
|  |  |  |  | UP8 - Innovation technology | 2.0 |
|  |  | W_Z_ (D2) = 13.9 | Safety performance  (SP) | SP1 - Structural bearing capacity | 2.5 |
|  |  |  |  | SP2 - Fire protection design of the existing building | 2.5 |
|  |  |  |  | SP3 - Safety of gas and electrical equipment | 2.4 |
|  |  |  |  | SP4 - Daily security precautions | 2.3 |
|  |  |  |  | SP5 - Indoor pollutant indicators | 2.2 |
|  |  |  |  | SP6 - Carbon emissions | 2.0 |
|  |  | W_Z_ (D3) = 18.2 | Durability performance (DP) | DP1 - Structural engineering performance | 2.3 |
|  |  |  |  | DP2 - Underground waterproof engineering | 2.4 |
|  |  |  |  | DP3 - Water-proof and moisture-proof of the room | 2.3 |
|  |  |  |  | DP4 - Roof waterproof construction | 2.4 |
|  |  |  |  | DP5 - Convenience of interior decoration | 2.0 |
|  |  |  |  | DP6 - Pipeline engineering | 2.3 |
|  |  |  |  | DP7 - Plant engineering | 2.3 |
|  |  |  |  | DP8 - Doors and windows performance | 2.2 |
| C2 | Economy  (12.9) | W_Z_ (D4) = 12.9 | Economic performance (ECP) | ECP1 - Energy saving | 2.3 |
|  |  |  |  | ECP2 - Water saving | 2.2 |
|  |  |  |  | ECP3 - Land saving | 1.9 |
|  |  |  |  | ECP4 - Material saving | 2.0 |
|  |  |  |  | ECP5 - Renewable energy utilization | 2.2 |
|  |  |  |  | ECP6 - Solid waste disposal | 2.3 |
| C3 | Environment  (26.6) | W_Z_ (D5) = 14 | Environmental livability (EL) | EL1 - Land use and planning | 2.0 |
|  |  |  |  | EL2 - Architectural facade style | 1.9 |
|  |  |  |  | EL3 - Green spaces and activity site | 2.3 |
|  |  |  |  | EL4 - Hydrographic net | 2.3 |
|  |  |  |  | EL5 - Natural landscape and diversity | 2.1 |
|  |  |  |  | EL6 - Historical relics and historical sites | 1.7 |
|  |  |  |  | EL7 - Humane landscapes | 1.7 |
|  |  | W_Z_ (D6) =12.6 | Indoor environmental quality | IEQ1 - Indoor pollutant indicators | 2.3 |
|  |  |  |  | IEQ2 - Control of greenhouse gases (GHS) emission sources | 1.7 |
|  |  |  |  | IEQ3 - Adequate flesh air | 2.2 |
|  |  |  |  | IEQ4 - Environmental tobacco smoke control | 2.1 |
|  |  |  |  | IEQ5 - Indoor environmental quality management | 2.1 |
|  |  |  |  | IEQ6 - Reduction of light pollution | 2.2 |
| C4 | Society  (13) | W_Z_ (D7) = 13.3 | Health and wellbeing (HW) | HW1 - Acoustic comfort | 2.2 |
|  |  |  |  | HW2 - Thermal comfort | 2.2 |
|  |  |  |  | HW3 - Natural ventilation | 2.0 |
|  |  |  |  | HW4 - Good daylighting and landscape | 2.2 |
|  |  |  |  | HW5 - Exterior noise and air contamination | 2.4 |
|  |  |  |  | HW6 - Outdoor environmental quality | 2.3 |

**Table S4**. Determination of credit points based a five-point scale.

| The second level index | The second–level judgement matrix: code | The third–level judgement matrix: code and index | Credit points (CP) |
| --- | --- | --- | --- |
| **W_z_ (D1) = 28.51** | **D1** | **Usage performance (UP)** | **28.51** |
|  |  | UP1 - Plane adaptability | 3.43 |
|  |  | UP2 - Intelligent facilities | 3.84 |
|  |  | UP3 - Building decoration | 2.62 |
|  |  | UP4 - Sound insulation performance | 3.99 |
|  |  | UP5 - Equipment and facility convenience | 3.58 |
|  |  | UP6 - Accessibility infrastructure and suitable for aging | 3.11 |
|  |  | UP7 - Transportation convenience | 4.14 |
|  |  | UP8 - Innovation technology | 3.8 |
| **W_z_ (D2) = 26.50** | **D3** | **Safety performance (SP)** | **26.5** |
|  |  | SP1 - Structural bearing capacity | 4.77 |
|  |  | SP2 - Fire protection design of the existing building | 4.72 |
|  |  | SP3 - Safety of gas and electrical equipment | 4.68 |
|  |  | SP4 - Daily security precautions | 4.31 |
|  |  | SP5 - Indoor pollutant indicators | 4.28 |
|  |  | SP6 - Carbon emissions | 3.74 |
| **W_z_ (D3) = 34.63** | **D4** | **Durability performance (DP)** | **34.63** |
|  |  | DP1 - Structural engineering performance | 4.28 |
|  |  | DP2 - Underground waterproof engineering | 4.5 |
|  |  | DP3 - Water-proof and moisture-proof of the room | 4.38 |
|  |  | DP4 - Roof waterproof construction | 4.62 |
|  |  | DP5 - Convenience of interior decoration | 3.95 |
|  |  | DP6 - Pipeline engineering | 4.36 |
|  |  | DP7 - Plant engineering | 4.3 |
|  |  | DP8 - Doors and windows performance | 4.24 |
| **W_z_ (D4) = 24.45** | **D4** | **Economic performance (ECP)** | **24.45** |
|  |  | ECP1 - Energy saving | 4.35 |
|  |  | ECP2 - Water saving | 4.24 |
|  |  | ECP3 - Land saving | 3.61 |
|  |  | ECP4 - Material saving | 3.82 |
|  |  | ECP5 - Renewable energy utilization | 4.11 |
|  |  | ECP6 - Solid waste disposal | 4.32 |
| **W_z_ (D5) = 26.69** | **D5** | **Environmental livability (EL)** | **26.69** |
|  |  | EL1 - Land use and planning | 3.78 |
|  |  | EL2 - Architectural facade style | 3.59 |
|  |  | EL3 - Green spaces and activity site | 4.42 |
|  |  | EL4 - Hydrographic net | 4.34 |
|  |  | EL5 - Natural landscape and diversity | 3.96 |
|  |  | EL6 - Historical relics and historical sites | 3.28 |
|  |  | EL7 - Humane landscapes | 3.32 |
| **W_z_ (D6) =24.06** | **D6** | **Indoor environmental quality (IEQ)** | **24.06** |
|  |  | IEQ1 - Indoor pollutant indicators | 4.38 |
|  |  | IEQ2 - Control of greenhouse gases (GHS) emission sources | 3.14 |
|  |  | IEQ3 - Adequate flesh air | 4.25 |
|  |  | IEQ4 - Environmental tobacco smoke control | 4.11 |
|  |  | IEQ5 - Indoor environmental quality management | 4.02 |
|  |  | IEQ6 - Reduction of light pollution | 4.16 |
| **W_z_ (D7) = 25.38** | **D7** | **Health and wellbeing (HW)** | **25.38** |
|  |  | HW1 - Acoustic comfort | 4.25 |
|  |  | HW2 - Thermal comfort | 4.11 |
|  |  | HW3 - Natural ventilation | 4.02 |
|  |  | HW4 - Good daylighting and landscape | 4.16 |
|  |  | HW5 - Exterior noise and air contamination | 4.49 |
|  |  | HW6 - Outdoor environmental quality | 4.35 |

**Table S5.** Hierarchical matrix for the performance assessment of teaching buildings of a middle school in Taiyuan, Shanxi Province, China.

| The second level index | The two–level judgement matrix: code | The third–level judgement matrix: code and index | Credit point (CP) |
| --- | --- | --- | --- |
| **W_z_ (D1) = 15.0** | **D1** | **Usage performance (UP)** | **13.6** |
|  |  | UP1 - Plane adaptability | 1.7 |
|  |  | UP2 - Intelligent facilities | 1.8 |
|  |  | UP3 - Building decoration | 1.2 |
|  |  | UP4 - Sound insulation performance | 1.9 |
|  |  | UP5 - Equipment and facility convenience | 1.7 |
|  |  | UP6 - Accessibility infrastructure and suitable for aging | 1.5 |
|  |  | UP7 - Transportation convenience | 1.7 |
|  |  | UP8 - Innovation technology | 2.1 |
| **W_z_ (D2) = 13.9** | **D3** | **Safety performance (SP)** | **11.5** |
|  |  | SP1 - Structural bearing capacity | 1.9 |
|  |  | SP2 - Fire protection design of the existing building | 2.3 |
|  |  | SP3 - Safety of gas and electrical equipment | 1.8 |
|  |  | SP4 - Daily security precautions | 1.8 |
|  |  | SP5 - Indoor pollutant indicators | 2.0 |
|  |  | SP6 - Carbon emissions | 1.7 |
| **W_z_ (D3) = 18.2** | **D4** | **Durability performance (DP)** | **15.6** |
|  |  | DP1 - Structural engineering performance | 2.1 |
|  |  | DP2 - Underground waterproof engineering | 2.2 |
|  |  | DP3 - Water-proof and moisture-proof of the room | 2.0 |
|  |  | DP4 - Roof waterproof construction | 2.0 |
|  |  | DP5 - Convenience of interior decoration | 1.9 |
|  |  | DP6 - Pipeline engineering | 1.8 |
|  |  | DP7 - Plant engineering | 1.7 |
|  |  | DP8 - Doors and windows performance | 1.9 |
| **W_z_ (D4) = 12.9** | **D4** | **Economic performance (ECP)** | **11.2** |
|  |  | ECP1 - Energy saving | 1.8 |
|  |  | ECP2 - Water saving | 1.9 |
|  |  | ECP3 - Land saving | 1.8 |
|  |  | ECP4 - Material saving | 1.8 |
|  |  | ECP5 - Renewable energy utilization | 2.0 |
|  |  | ECP6 - Solid waste disposal | 1.9 |
| **W_z_ (D5) = 14.0** | **D5** | **Environmental livability (EL)** | **11.7** |
|  |  | EL1 - Land use and planning | 1.9 |
|  |  | EL2 - Architectural facade style | 1.6 |
|  |  | EL3 - Green spaces and activity site | 2.1 |
|  |  | EL4 - Hydrographic net | 1.8 |
|  |  | EL5 - Natural landscape and diversity | 2.0 |
|  |  | EL6 - Historical relics and historical sites | 1.0 |
|  |  | EL7 - Humane landscapes | 1.3 |
| **W_z_ (D6) = 12.6** | **D6** | **Indoor environmental quality (IEQ)** | **10.0** |
|  |  | IEQ1 - Indoor pollutant indicators | 2.0 |
|  |  | IEQ2 - Control of greenhouse gases (GHS) emission sources | 1.6 |
|  |  | IEQ3 - Adequate flesh air | 1.7 |
|  |  | IEQ4 - Environmental tobacco smoke control | 1.6 |
|  |  | IEQ5 - Indoor environmental quality management | 1.5 |
|  |  | IEQ6 - Reduction of light pollution | 1.6 |
| **W_z_ (D7) = 13.3** | **D7** | **Health and wellbeing (HW)** | **11.2** |
|  |  | HW1 - Acoustic comfort | 1.7 |
|  |  | HW2 - Thermal comfort | 1.8 |
|  |  | HW3 - Natural ventilation | 1.9 |
|  |  | HW4 - Good daylighting and landscape | 1.8 |
|  |  | HW5 - Exterior noise and air contamination | 2.0 |
|  |  | HW6 - Outdoor environmental quality | 2.0 |

**Appendix A.** Questionnaire of ranking second level indicators.

Please rate the indicators with the 12 options.

|  | Extreme important | Very strong important | Strong important | Moderate important | Equal unimportant | Slightly important | Slightly unimportant | Moderate unimportant | Strong unimportant | Very strong unimportant | Extreme unimportant | Not at all |
| --- | --- | --- | --- | --- | --- | --- | --- | --- | --- | --- | --- | --- |
|  | 1 | 2 | 3 | 4 | 5 | 6 | 7 | 8 | 9 | 10 | 11 | 12 |
| **Usage** | □ | □ | □ | □ | □ | □ | □ | □ | □ | □ | □ | □ |
| **Environment livability** | □ | □ | □ | □ | □ | □ | □ | □ | □ | □ | □ | □ |
| **Economy** | □ | □ | □ | □ | □ | □ | □ | □ | □ | □ | □ | □ |
| **Safety** | □ | □ | □ | □ | □ | □ | □ | □ | □ | □ | □ | □ |
| **Durability** | □ | □ | □ | □ | □ | □ | □ | □ | □ | □ | □ | □ |
| **Manage** | □ | □ | □ | □ | □ | □ | □ | □ | □ | □ | □ | □ |
| **Indoor environmental quality** | □ | □ | □ | □ | □ | □ | □ | □ | □ | □ | □ | □ |
| **Health and wellbeing** | □ | □ | □ | □ | □ | □ | □ | □ | □ | □ | □ | □ |
| **Carbon emissions** | □ | □ | □ | □ | □ | □ | □ | □ | □ | □ | □ | □ |
| **Innovation techniques** | □ | □ | □ | □ | □ | □ | □ | □ | □ | □ | □ | □ |
| **Convenience** | □ | □ | □ | □ | □ | □ | □ | □ | □ | □ | □ | □ |
| **Comfort** | □ | □ | □ | □ | □ | □ | □ | □ | □ | □ | □ | □ |

**Appendix B.** Questionnaire of the third level indicators.

Please rate the level of significance of the sub-indicators with the 5 options.

|  | Negligible | Optional | Common | Required | Mandatory |
| --- | --- | --- | --- | --- | --- |
| **Usage performance (UP)** |  |  |  |  |  |
| UP1 - Plane adaptability | □ | □ | □ | □ | □ |
| UP2 - Intelligent facilities | □ | □ | □ | □ | □ |
| UP3 - Building decoration | □ | □ | □ | □ | □ |
| UP4 - Sound insulation performance | □ | □ | □ | □ | □ |
| UP5 - Equipment and facility convenience | □ | □ | □ | □ | □ |
| UP6 - Accessibility infrastructure and suitable for aging | □ | □ | □ | □ | □ |
| UP7 - Transportation convenience | □ | □ | □ | □ | □ |
| UP8 - Innovation technology | □ | □ | □ | □ | □ |
| **Safety performance (SP)** |  |  |  |  |  |
| SP1 - Structural bearing capacity | □ | □ | □ | □ | □ |
| SP2 - Fire protection design of the existing building | □ | □ | □ | □ | □ |
| SP3 - Safety of gas and electrical equipment | □ | □ | □ | □ | □ |
| SP4 - Daily security precautions | □ | □ | □ | □ | □ |
| SP5 - Indoor pollutant indicators | □ | □ | □ | □ | □ |
| SP6 - Carbon emissions | □ | □ | □ | □ | □ |
| **Durability performance (DP)** | □ | □ | □ | □ | □ |
| DP1 - Structural engineering performance |  |  |  |  |  |
| DP2 - Underground waterproof engineering | □ | □ | □ | □ | □ |
| DP3 - Water-proof and moisture-proof of the room | □ | □ | □ | □ | □ |
| DP4 - Roof waterproof construction | □ | □ | □ | □ | □ |
| DP5 - Convenience of interior decoration | □ | □ | □ | □ | □ |
| DP6 - Pipeline engineering | □ | □ | □ | □ | □ |
| DP7 - Plant engineering | □ | □ | □ | □ | □ |
| DP8 - Doors and windows performance | □ | □ | □ | □ | □ |
| **Economic performance (ECP)** |  |  |  |  |  |
| ECP1 - Energy saving | □ | □ | □ | □ | □ |
| ECP2 - Water saving | □ | □ | □ | □ | □ |
| ECP3 - Land saving | □ | □ | □ | □ | □ |
| ECP4 - Material saving | □ | □ | □ | □ | □ |
| ECP5 - Renewable energy utilization | □ | □ | □ | □ | □ |
| ECP6 - Solid waste disposal | □ | □ | □ | □ | □ |
| **Environmental livability (EL)** |  |  |  |  |  |
| EL1 - Land use and planning | □ | □ | □ | □ | □ |
| EL2 - Architectural facade style | □ | □ | □ | □ | □ |
| EL3 - Green spaces and activity site | □ | □ | □ | □ | □ |
| EL4 - Hydrographic net | □ | □ | □ | □ | □ |
| EL5 - Natural landscape and diversity | □ | □ | □ | □ | □ |
| EL6 - Historical relics and historical sites | □ | □ | □ | □ | □ |
| EL7 - Humane landscapes | □ | □ | □ | □ | □ |
| **Indoor environmental quality (IEQ)** |  |  |  |  |  |
| IEQ1 - Indoor pollutant indicators | □ | □ | □ | □ | □ |
| IEQ2 - Control of greenhouse gases (GHS) emission sources | □ | □ | □ | □ | □ |
| IEQ3 - Adequate flesh air | □ | □ | □ | □ | □ |
| IEQ4 - Environmental tobacco smoke control | □ | □ | □ | □ | □ |
| IEQ5 - Indoor environmental quality management | □ | □ | □ | □ | □ |
| IEQ6 - Reduction of light pollution | □ | □ | □ | □ | □ |
| **Health and wellbeing (HW)** |  |  |  |  |  |
| HW1 - Acoustic comfort | □ | □ | □ | □ | □ |
| HW2 - Thermal comfort | □ | □ | □ | □ | □ |
| HW3 - Natural ventilation | □ | □ | □ | □ | □ |
| HW4 - Good daylighting and landscape | □ | □ | □ | □ | □ |
| HW5 - Exterior noise and air contamination | □ | □ | □ | □ | □ |
| HW6 - Outdoor environmental quality | □ | □ | □ | □ | □ |

**Appendix C.** Questionnaire of the third level indicators.

Please rank the indicators by making a selection from the 5 options.

|  | Equal unimportant  1 score | Moderate important  2 score | Strong important  3 score | Very strong important  4 score | Extreme important  5 score |
| --- | --- | --- | --- | --- | --- |
| **Usage performance (UP)** |  |  |  |  |  |
| UP1 - Plane adaptability | □ | □ | □ | □ | □ |
| UP2 - Intelligent facilities | □ | □ | □ | □ | □ |
| UP3 - Building decoration | □ | □ | □ | □ | □ |
| UP4 - Sound insulation performance | □ | □ | □ | □ | □ |
| UP5 - Equipment and facility convenience | □ | □ | □ | □ | □ |
| UP6 - Accessibility infrastructure and suitable for aging | □ | □ | □ | □ | □ |
| UP7 - Transportation convenience | □ | □ | □ | □ | □ |
| UP8 - Innovation technology | □ | □ | □ | □ | □ |
| **Safety performance (SP)** |  |  |  |  |  |
| SP1 - Structural bearing capacity | □ | □ | □ | □ | □ |
| SP2 - Fire protection design of the existing building | □ | □ | □ | □ | □ |
| SP3 - Safety of gas and electrical equipment | □ | □ | □ | □ | □ |
| SP4 - Daily security precautions | □ | □ | □ | □ | □ |
| SP5 - Indoor pollutant indicators | □ | □ | □ | □ | □ |
| SP6 - Carbon emissions | □ | □ | □ | □ | □ |
| **Durability performance (DP)** | □ | □ | □ | □ | □ |
| DP1 - Structural engineering performance |  |  |  |  |  |
| DP2 - Underground waterproof engineering | □ | □ | □ | □ | □ |
| DP3 - Water-proof and moisture-proof of the room | □ | □ | □ | □ | □ |
| DP4 - Roof waterproof construction | □ | □ | □ | □ | □ |
| DP5 - Convenience of interior decoration | □ | □ | □ | □ | □ |
| DP6 - Pipeline engineering | □ | □ | □ | □ | □ |
| DP7 - Plant engineering | □ | □ | □ | □ | □ |
| DP8 - Doors and windows performance | □ | □ | □ | □ | □ |
| **Economic performance (ECP)** |  |  |  |  |  |
| ECP1 - Energy saving | □ | □ | □ | □ | □ |
| ECP2 - Water saving | □ | □ | □ | □ | □ |
| ECP3 - Land saving | □ | □ | □ | □ | □ |
| ECP4 - Material saving | □ | □ | □ | □ | □ |
| ECP5 - Renewable energy utilization | □ | □ | □ | □ | □ |
| ECP6 - Solid waste disposal | □ | □ | □ | □ | □ |
| **Environmental livability (EL)** |  |  |  |  |  |
| EL1 - Land use and planning | □ | □ | □ | □ | □ |
| EL2 - Architectural facade style | □ | □ | □ | □ | □ |
| EL3 - Green spaces and activity site | □ | □ | □ | □ | □ |
| EL4 - Hydrographic net | □ | □ | □ | □ | □ |
| EL5 - Natural landscape and diversity | □ | □ | □ | □ | □ |
| EL6 - Historical relics and historical sites | □ | □ | □ | □ | □ |
| EL7 - Humane landscapes | □ | □ | □ | □ | □ |
| **Indoor environmental quality (IEQ)** |  |  |  |  |  |
| IEQ1 - Indoor pollutant indicators | □ | □ | □ | □ | □ |
| IEQ2 - Control of greenhouse gases (GHS) emission sources | □ | □ | □ | □ | □ |
| IEQ3 - Adequate flesh air | □ | □ | □ | □ | □ |
| IEQ4 - Environmental tobacco smoke control | □ | □ | □ | □ | □ |
| IEQ5 - Indoor environmental quality management | □ | □ | □ | □ | □ |
| IEQ6 - Reduction of light pollution | □ | □ | □ | □ | □ |
| **Health and wellbeing (HW)** |  |  |  |  |  |
| HW1 - Acoustic comfort | □ | □ | □ | □ | □ |
| HW2 - Thermal comfort | □ | □ | □ | □ | □ |
| HW3 - Natural ventilation | □ | □ | □ | □ | □ |
| HW4 - Good daylighting and landscape | □ | □ | □ | □ | □ |
| HW5 - Exterior noise and air contamination | □ | □ | □ | □ | □ |
| HW6 - Outdoor environmental quality | □ | □ | □ | □ | □ |
